# On the Utility of ToxCast™ and ToxPi as Methods for Identifying New Obesogens

**DOI:** 10.1289/ehp.1510352

**Published:** 2016-01-13

**Authors:** Amanda Shaine Janesick, Giorgio Dimastrogiovanni, Lenka Vanek, Christy Boulos, Raquel Chamorro-García, Weiyi Tang, Bruce Blumberg

**Affiliations:** 1 Department of Developmental and Cell Biology, University of California, Irvine, Irvine, California, USA; 2 Department of Environmental Chemistry, IIQAB-CSIC (Superior Council of Scientific Investigations), Barcelona, Spain; 3 Department of Pharmaceutical Sciences, University of California, Irvine, Irvine, California, USA

## Abstract

**Background:**

In ToxCast™ Phase I, the U.S. EPA commissioned screening of 320 pesticides, herbicides, fungicides, and other chemicals in a series of high-throughput assays. The agency also developed a toxicological prioritization tool, ToxPi, to facilitate using ToxCast™ assays to predict biological function.

**Objectives:**

We asked whether top-scoring PPARγ activators identified in ToxCast™ Phase I were genuine PPARγ activators and inducers of adipogenesis. Next, we identified ToxCast™ assays that should predict adipogenesis, developed an adipogenesis ToxPi, and asked how well the ToxPi predicted adipogenic activity.

**Methods:**

We used transient transfection to test the ability of ToxCast™ chemicals to modulate PPARγ and RXRα, and differentiation assays employing 3T3-L1 preadipocytes and mouse bone marrow-derived mesenchymal stem cells (mBMSCs) to evaluate the adipogenic capacity of ToxCast™ chemicals.

**Results:**

Only 5/21 of the top scoring ToxCast™ PPARγ activators were activators in our assays, 3 were PPARγ antagonists, the remainder were inactive. The bona fide PPARγ activators we identified induced adipogenesis in 3T3-L1 cells and mBMSCs. Only 7 of the 17 chemicals predicted to be active by the ToxPi promoted adipogenesis, 1 inhibited adipogenesis, and 2 of the 7 predicted negatives were also adipogenic. Of these 9 adipogenic chemicals, 3 activated PPARγ, and 1 activated RXRα.

**Conclusions:**

ToxCast™ PPARγ and RXRα assays do not correlate well with laboratory measurements of PPARγ and RXRα activity. The adipogenesis ToxPi performed poorly, perhaps due to the performance of ToxCast™ assays. We observed a modest predictive value of ToxCast™ for PPARγ and RXRα activation and adipogenesis and it is likely that many obesogenic chemicals remain to be identified.

**Citation:**

Janesick AS, Dimastrogiovanni G, Vanek L, Boulos C, Chamorro-García R, Tang W, Blumberg B. 2016. On the utility of ToxCast™ and ToxPi as methods for identifying new obesogens. Environ Health Perspect 124:1214–1226; http://dx.doi.org/10.1289/ehp.1510352

## Introduction

In 1996, the Food Quality Protection Act (FQPA 1996) and the Safe Drinking Water Act Amendments (SDWA Amendments 1996) directed the U.S. Environmental Protection Agency (EPA) to develop a screening program that would identify endocrine-disrupting chemicals (EDCs) targeting the androgen, estrogen, and thyroid signaling pathways. One key outcome is that the U.S. EPA developed the Toxicity Forecaster (ToxCast™) program in 2007 (Dix et al. 2007). The stated goal of ToxCast™ was to employ high-throughput screening (HTS) assays to prioritize chemicals and use this information to inform regulatory decisions regarding thousands of environmental contaminants (Dix et al. 2007). The rationale was that a vanishingly small number of chemicals had been tested adequately for toxicity, and even fewer for endocrine-disrupting end points. Currently, 8 million unique organic compounds are available for purchase (Chuprina et al. 2010), and approximately 84,000 chemicals are registered with the U.S. EPA under the Toxic Substances Control Act of 1976 (TSCA 1976). The U.S. EPA Chemical Data Reporting revealed that over 7,000 chemicals are in wide use (annual production volume > 100,000 lbs) (U.S. EPA 2014b). Other estimates that include data sources from the United States, Canada, and Europe conclude that 30,000 chemicals are in wide commercial use (> 1 ton/year) (Muir and Howard 2006). Health and toxicity data for most chemicals remains elusive because TSCA grandfathered tens of thousands of chemicals that were already on the market before 1976, none of which underwent U.S. EPA review and for which scant safety data are available.

In 2007, the National Research Council recommended *in vitro* assays to determine which toxicity pathways contribute to human disease (Collins et al. 2008; Kavlock et al. 2009). As a result, ToxCast™ implemented Phase 1 *in vitro* testing. ToxCast™ Phase 1 was a proof-of-concept study whereby 320 pesticides, mostly agrochemicals, were selected based on historical toxicological evidence, including *in vivo* carcinogenicity, reproductive, and developmental defects (Dix et al. 2007). Phase 1 chemicals were subjected to > 450 assays and prioritized by cluster and discriminant analysis using multiple inputs: *in silico* predictions from physicochemical properties, radioligand/enzyme biochemistry, transcription reporter assays, microarray, cytotoxicity, cell growth kinetics, and more (Dix et al. 2007). In Phase 2, 700 additional chemicals (for which toxicological data is more sparse compared to Phase 1) were tested (Kavlock et al. 2012). Since its 2007 inception, ToxCast™ has been reformulated as a prescreening effort to the U.S. EPA Endocrine Disruptor Screening Program (EDSP) to prioritize chemicals for subsequent, *in vivo* testing.

The peroxisome proliferator–activated receptor gamma (PPARγ) is a key regulator of adipogenesis (Tontonoz and Spiegelman 2008). PPARγ heterodimerizes with the 9-cis retinoic acid receptor (RXR) and directly promotes transcription of such key adipogenic genes as fatty acid binding protein 4 (*Fabp4*), lipoprotein lipase (*Lpl*) and adiponectin (*Adipoq*) (Tontonoz and Spiegelman 2008). Some environmental EDCs activate PPARγ and RXR, thereby promoting adipogenesis, whereas others promote adipogenesis by as yet unknown pathways (Janesick and Blumberg 2011b). These obesogens typically act at low, environmentally relevant doses [often below the established no-observed-adverse-effect-level, (NOAEL)] during critical windows of prenatal or postnatal development to promote obesity later in life (Grün and Blumberg 2006; Janesick and Blumberg 2011a). Obesogens can also alter the epigenetic memory of cells, creating lasting, transgenerational effects on obesity and metabolic end points (Chamorro-García and Blumberg 2014; Chamorro-García et al. 2013; Janesick et al. 2014).

When we began this project, there were no published studies investigating the reliability of ToxCast™ assays. Subsequently, U.S. EPA scientists have evaluated the performance of estrogen and androgen assays as pre-screens for chemicals to be further tested in the U.S. EPA EDSP (Reif et al. 2010; Rotroff et al. 2013). Since several ToxCast™ assays measure the ability of chemicals to bind to, or activate PPARγ, we first sought to test how reliable the assays (performed by commercial contractors) were in a laboratory setting. Next, prompted by a meeting hosted by the National Institute of Environmental Health Sciences (NIEHS) to evaluate the evidence for the involvement of EDCs in obesity and diabetes (Thayer et al. 2012), we identified a set of ToxCast™ assays that should predict the adipogenic potential of chemicals. These assays were used to generate a toxicological priority index (ToxPi) (Reif et al. 2010) that we expected to predict the ability of chemicals to promote adipogenesis in cell culture models. In principle, ToxCast™ assays and ToxPi should be useful tools for identifying chemicals that target various adverse outcome pathways. However, we show here that the results of ToxCast™ PPARγ and RXRα assays do not correlate well with activity measured in a laboratory setting and that there is little agreement among ToxCast™ assays on the same end points. We further found that the ToxPi we designed for adipogenesis performed poorly in identifying potential obesogens and that the results were rife with false positives. Despite the poor overall performance of ToxCast™ assays and the ToxPi, some obesogens and potential obesogens were identified. We expect that if poorly performing ToxCast™ assays were improved (or replaced) the utility of ToxCast™ and ToxPi could be improved markedly and the promise of this important program realized.

## Methods

### ToxCast™ Phase 1 Assays

We used publically available data from three main assays reported in ToxCast™ Phase 1: Attagene Factorial™ Transcription Reporter System, National Institutes of Health (NIH) Chemical Genomics Center (NCGC) Invitrogen™ GeneBLAzer^®^ technology, and NovaScreen® Direct Binding (see Table S1). Attagene Factorial™ is a high-throughput assay that uses capillary gel electrophoresis to track multiple reporters within the same population of transiently transfected cells simultaneously (Romanov et al. 2008). Trans-Factorial™ assays use receptor ligand-binding domains (LBD) fused to the GAL4 DNA-binding domain (DBD), whereas Cis Factorial™ assays use identified nuclear hormone receptor response elements without added receptors (Romanov et al. 2008). NovaScreen® [PerkinElmer (formerly Caliper Life Sciences)] uses fluorescence polarization (Jameson and Sawyer 1995; Jolley et al. 1981) or scintillation proximity (Sweetnam et al. 1993) technology to detect binding of chemicals to hPPARγ in competition with fluorescent ciglitazone, or binding to human glucocorticoid receptor (hGR) in competition with [^3^H]-dexamethasone. These binding assays cannot differentiate whether a chemical is an activator or antagonist of a receptor, but measure apparent binding affinity, *in vitro*. NCGC GeneBLAzer^®^ technology (Invitrogen™), utilizes a GAL4 DBD nuclear receptor LBD, GAL4_UAS_ *β-lactamase* reporter, and a FRET-based substrate, which creates blue color when modified by β-lactamase (Knight et al. 2009; Zlokarnik et al. 1998). We tested the top 20 ranked activators of PPARγ from ToxCast™ Phase 1 (see Table S2). These chemicals were supplied by the National Toxicology Program (NTP) from the same stocks that were utilized in ToxCast™ Phase 1. We also included chlorothalonil, which the NovaScreen® PPARγ direct binding assay indicated bound strongly to PPARγ (see Table S2). For analysis of the ToxPi, all chemicals tested were supplied by NTP and derived from ToxPi scoring of 16 different assays (see Tables S1 and S3), which is explained in further detail in the next section (“Phase I ToxPi Construction”).

### Phase I ToxPi Construction

We supplied a list of gene targets to the NIH/NIEHS (K. Thayer) that the scientific literature and experience suggest could be useful to predict adipogenesis. Sixteen assays from Attagene, NovaScreen®, and NCGC (see Table S1) interrogated these targets and were incorporated into ToxPi models that were constructed by two NIEHS/NTP scientists (D. Reif and V. Walker) (see Table S3). These 16 assays were chosen because they were relevant to the biological process of adipogenesis. Three out of sixteen assays showed no activation by any of the 320 ToxCast™ chemicals. ToxPi scores and rankings were achieved using analysis previously published (Filer et al. 2014; Reif et al. 2010, 2013). Briefly, each slice of the ToxPi is composed of one assay or a collection of assays. For example, the LXRE slice is one assay (Attagene, DR4-CIS assay), but the PPARγ slice represents 3 assays (see Table S1). Scores and rankings were generated by summing the AC_50_ values of the assays within each slice for each chemical. Highly ranked chemicals either have very low AC_50_ values for 1–2 assays, or moderately low AC_50_ values across many assays (see Table S3). To generate the input data for the ToxPi analyses, we used AC_50_ values available in the 14 January 2011 ToxCast™ Phase I release (Knudsen et al. 2011). For any chemical where the AC_50_ was not applicable, the AC_50_ for that particular assay was set to 1,000,000 (1 Molar).

### Transient Transfection Analysis

pCMX*-Gal4*, pCMX*-Gal4*-*mPPARγ*, and pCMX*-Gal4-hRXRα* were previously described (Grün et al. 2006). Transient transfections were performed in COS7 cells as described (Chamorro-García et al. 2012). Briefly, COS7 cells were seeded at 15,000 cells per well in 96-well tissue culture plates in 10% calf bovine serum. The following day, cells were transfected in Opti-MEM at ~ 90% confluency. One microgram of CMX-GAL4 effector plasmid was co-transfected with 5 μg tk-(MH100)_4_-*luciferase* reporter and 5 μg of CMX-*β-galactosidase* transfection control plasmids in Opti-MEM® using Lipofectamine® 2000 reagent (Invitrogen™ Life Technologies, Carlsbad, CA, USA), following the manufacturer’s recommended protocol. All chemicals were solvated in dimethyl sulfoxide (DMSO). After overnight incubation, the medium was replaced with Dulbecco’s Modified Eagle Medium (DMEM; HyClone, Logan, UT, USA), 10% resin charcoal stripped fetal bovine serum (FBS) plus ligands at concentrations indicated in the figure legends for an additional 24 hr. DMSO concentration was maintained at 0.05% across all chemical treatments. Cells were lysed and assayed for luciferase and β-galactosidase activity as previously described (Forman et al. 1995). All transfections were performed in triplicate and reproduced in multiple experiments. Data are reported as fold induction or reduction over vehicle (0.1% DMSO) controls ± SEM (standard error of the mean) using standard propagation of error (Bevington and Robinson 2003). EC_50_ and IC_50_ values (half- maximal effective or inhibitory concentration) for the active chemicals were obtained using nonlinear regression, variable slope in GraphPad Prism 5.0 (Graphpad Software Inc., San Diego, CA) (see Figure S1). Spirodiclofen did not plateau; therefore, it was constrained at the top dose. EC_10_ and IC_10_ values (10% maximal effective or inhibitory concentration) were calculated in GraphPad Quick Calc (Compute EC_anything_ from EC_50_). The EC_50_, EC_10_, IC_50_, and IC_10_ values from NCGC and ToxCast™ are reported from gain AC_50_ and AC_10_ values in the ToxCast™ 2014 release (Filer et al. 2014).

### Adipogenesis Assays—Cell Culture (Figure S2)

3T3-L1 cells (ATCC) were maintained in high-glucose DMEM supplemented with 10% fetal bovine serum, 2 mM L-glutamine, 50 IU/mL penicillin, and 50 μg/mL streptomycin. A total of 2 × 10^4^ cells per well were seeded in 12-well plates. After 48 hr, cells were exposed to the adipogenic cocktail MDI (500 μM isobutylmethylxanthine, 0.25 nM dexamethasone and 5 μg/mL insulin), 8 μg/mL biotin and 8 μg/mL pantothenate for 2 days. Induction media was removed and cells were exposed to test chemicals during 5 days, replacing the media every 2 days. Rosiglitazone (ROSI) and tributyltin (TBT) were used as positive controls at 100 nM and 50 nM final concentrations, respectively. All ToxCast™ chemicals were tested at 0.02, 0.2, 2, and 20 μM, and DMSO concentration was maintained at 0.1% across all treatments. If the chemical was toxic at 20 μM, we repeated the experiment at 10 μM.

A total of 8 × 10^4^ cells/well mouse bone marrow-derived mesenchymal stem cells (mBMSCs) (OriCell™) were seeded in 12-well plates in basic medium: high-glucose DMEM (Hyclone) containing 10% calf bovine serum (Premium Select, Atlanta Biologicals), 100 IU/mL penicillin, 100 μg/mL streptomycin, and 1 mM sodium pyruvate. mBMSCs were induced to differentiate in differentiation media (low glucose αMEM containing 15% fetal bovine serum (Premium Select, Atlanta Biologicals), 100 IU/mL penicillin, 100 μg/mL streptomycin and 2 mM L-glutamine) with adipogenic cocktail (500 μM isometylbutylxanthine, 1 μM dexamethasone, 5 μg/mL insulin) and either 500 nM ROSI, 50 nM TBT or ToxCast™ chemicals (as noted for 3T3-L1 cells) for 14 days, replacing the media every 3 days.

### Adipogenesis Assays—Quantitation

Cells were either fixed in 3.7% formaldehyde in phosphate-buffered saline (PBS) for 30 min at room temperature (RT) for lipid quantitation, or homogenized in TriPure (Roche, Mannheim, Germany) for gene expression analysis. For lipid quantitation, fixed cells were washed twice with PBS and maintained in PBS overnight at 4°C to release residual phenol red. Background of cells were measured prior to staining. Cells were stained with 1 μg/mL Nile red (CAS No. 7385-67-3) (to detect lipid accumulation) and 1 μg/mL Hoechst 33342 (ThermoFisher Scientific) (to detect nuclei as a surrogate for cell number) in PBS for 15 min in the dark at RT and washed twice with PBS. RFUs (relative fluorescence units) were measured for Hoechst 33342 (355 excitation, 460 emission) and Nile red (485 excitation, 590 emission) in a SpectraMax Gemini XS 96-well spectrofluorometer (Molecular Devices, Sunnyvale, CA, USA). Background values of Hoechst and Nile red were subtracted from the RFUs after staining and the ratio RFU_Nile Red_:RFU_Hoechst_ was calculated. Data are represented as mean ± SEM. Total RNA was isolated using TriPure (Roche) as recommended by the manufacturer. Reverse transcription and quantitative real-time RT-PCR (QPCR) were performed using Transcriptor RT and SYBR® Green Master Mix (Roche). cDNA was quantitated in a LightCycler® 480 System (Roche Diagnostics, Basel, Switzerland) using primer sets listed in Table S4. Each primer set amplified a single band as determined by gel electrophoresis and melting curve analysis. QPCR data were analyzed using the 2^ΔΔCt^ method (Livak and Schmittgen 2001) relative to ribosomal protein *36b4*, normalizing to 0.1% DMSO vehicle. Error bars represent the SEM from four to six biological replicates calculated using standard propagation of error (Bevington and Robinson 2003).

### Phase II PPARγ Activators and ToxPi Construction

Phase II ToxCast™ data includes 1,858 chemicals (Filer et al. 2014) which have associated *Z-*score corrections for each chemical-assay pair. We obtained *Z-*scores and log(AC_50_) values from the ToxCast™ Data Summary Files (http://www.epa.gov/ncct/ToxCast™/data.html) for the 320 Phase I chemicals. *Z*-scores are a measurement of potency relative to cytotoxicity and are often employed to remove false positive chemicals (U.S. EPA 2014a). Phase II ToxPi diagrams were constructed using Phase II 2014 release data (Filer et al. 2014) with the Phase I chemical library. Recently, Phase II ToxPi diagrams have been constructed by first removing chemicals with low *Z*-scores, and then incorporating the *Z*-score into the magnitude of the Pi slice (Kris Thayer, personal communication). Following this method, we created new ToxPi diagrams either solely based on AC_50_ values, or by removing chemicals with cytotoxicity *Z-*scores less than 3 and then correcting the magnitude of the Pi slice by incorporating *Z-*scores. This was accomplished by converting the AC_50_ value to negative log molar units (e.g., 1 μM = 6), then adding the *Z-*score value. Final rankings were generated using the ToxPi algorithm (Reif et al. 2010). For re-evaluation of PPARγ activators using Phase II data for three PPARγ assays (Attagene, NVS, NCGC/Tox21) (see Table S5), the Phase I chemical library was ranked based solely on AC_50_ (half-maximal activity concentration) values, or by removing chemicals with cytotoxicity *Z-*scores > 3 (U.S. EPA 2014a) and then adding the *Z-*score to the AC_50_ as described above. Final rankings were generated using the ToxPi algorithm weighting all three PPARγ assays equally.

### Statistical Analysis

Statistical analysis and graphing was conducted in GraphPad Prism 5.0 (Graphpad Software Inc.). One-way ANOVA was used to determine differences in relative mRNA abundance or staining among ToxCast™ treatment groups and negative control (DMSO). This was followed by a Dunnett’s post hoc test to ascertain statistical significance for each ToxCast™ treatment group compared to the control (DMSO). An unpaired *t*-test was used to determine the significance of effects elicited by the positive controls, ROSI or TBT, relative to DMSO. We considered *p* ≤ 0.05 to be statistically significant.

## Results

### PPARγ Activation by Phase I ToxCast™ PPARγ Activators=

**Figure 1:**
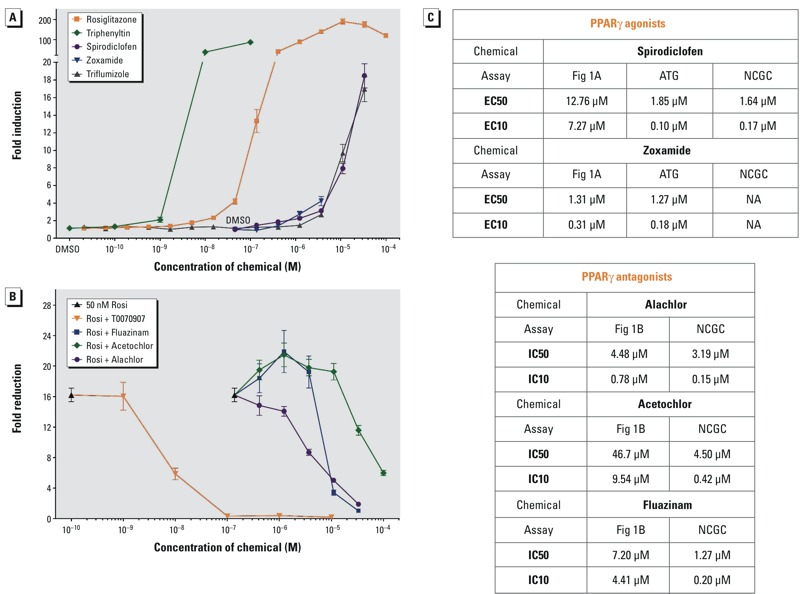
ToxCast^™^ Chemical Activity on PPARγ. The ability of a graded dose series of ToxCast^™^ chemicals to (*A*) activate or (*B*) antagonize GAL4-mPPARγ was tested in transiently transfected COS7 cells. (*A*, *B*) Data points are averages of triplicate transfections (three biological replicates). Cytotoxicity, as measured by decreased β-galactosidase activity was observed at 100 μM for spirodiclofen, triflumizole, alachlor, and fluazinam, ≥ 10 μM for zoxamide, and ≥ 1 μM for tri­phenyltin. Data are depicted as (*A*) fold induction or (*B*) reduction over vehicle (0.05% DMSO) controls ± SEM. (*A*) ToxCast^™^ chemicals were tested in 3-fold serial dilutions from 100 μM through 0.137 μM, with the final data point being 0.05% DMSO. Rosiglitazone serves as a positive control activator. (*B*) ToxCast^™^ chemicals were tested in 3-fold serial dilutions from 100 μM, in competition with 50 nM rosiglitazone (Rosi). T0070907 (2-chloro-5-nitro-N-4-pyridinylbenzamide) serves as a positive control PPARγ antagonist. (*C*) EC_50_, EC_10_, IC_50_, and IC_10_ values calculated from *A* and *B* are reported and compared to commercial assays (see Figure S1). Note: ATG, Attagene GAL-PPARγ activation assay; NCGC, GeneBLAzer^®^ agonist (EC values) or antagonist (IC values) assays. Triphenyltin was previously published (Kanayama et al. 2005).

Three NIEHS/NTP scientists (K. Thayer, D. Reif, and V. Walker) provided us with the 20 highest ranked activators of PPARγ from ToxCast™ Phase I. AC_50_ values were largely driven by the Attagene Gal-PPARγ trans-Factorial™ Transcription Reporter assays (Martin et al. 2010) (see Table S1). An additional chemical, chlorothalonil, was negative in Attagene assays but was reported to bind avidly (AC_50_ = 0.6 μM) in the NovaScreen® PPARγ ligand-binding assay. We tested these activators and chlorothalonil in Cos7 cells using transient transfection assays and found that only 4 out of 21 chemicals (spirodiclofen, zoxamide, triphenyltin, and triflumizole) activated GAL-PPARγ (Figure 1A). Of the reported PPARγ agonists, 3 out of 20 (fluazinam, alachlor, and acetochlor) did not activate PPARγ; rather, they were weak antagonists in competition assays against 50 nM rosiglitazone (Figure 1B). The GAL4 DBD alone was not activated or repressed by any of the chemicals tested or the controls (data not shown). Quinoxyfen was subsequently identified as weakly active at 100 μM and 33 μM doses in the ToxPI analysis discussed later in this manuscript [see “Selection of Assays for ToxPi Construction” (Figure 4A)]. The EC_50_, EC_10_, IC_50_, and IC_10_ values for the active chemicals were calculated, reported, and compared to Attagene and NCGC/Tox21 assay data (release 2014) (Filer et al. 2014) in Figure 1C. Subsequent to our studies, U.S. EPA scientists have re-evaluated the results of ToxCast™ Phase 1 with respect to non-specific induction of reporter gene activity in some assays and tested additional chemicals to yield ToxCast™ Phase 2 (Kavlock et al. 2012).

### Effects of Spirodiclofen and Zoxamide on Adipogenesis in Cell Culture Models

**Figure 2:**
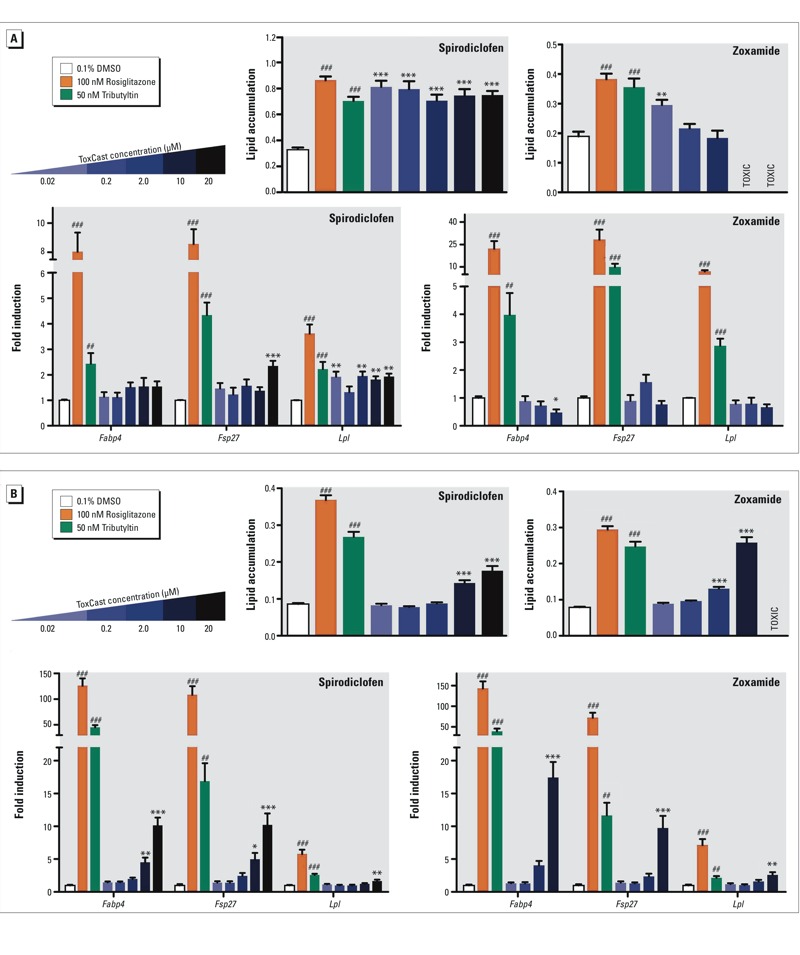
ToxCast^™^ chemicals zoxamide and spirodiclofen induce adipogenesis in 3T3-L1 cells and mouse bone marrow-derived mesenchymal stem cells (mBMSCs). Adipogenesis was induced in cells according to Figure S2. Lipid accumulation was quantified in differentiated (*A*) 3T3-L1 preadipocytes or (*B*) mBMSCs by measuring Nile red fluorescence normalized by cell number (Hoechst). Rosiglitazone and tributyltin serve as positive control adipogenic chemicals. Gene expression was determined by the 2^–^^ΔΔ^ ^CT^ method using *36b4* as the reference gene. Data are reported as fold induction over 0.1% DMSO vehicle controls ± SEM using standard propagation of error. Primer sequences can be found in Table S4. One-way ANOVA was conducted for zoxamide and spirodiclofen treatment groups and DMSO vehicle, followed by Dunnett’s post hoc test: *p ≤ 0.05, **p ≤ 0.01, ***p ≤ 0.001 compared to vehicle. Unpaired *t*-test was conducted for the positive controls rosiglitazone, tributyltin versus vehicle: ^#^ *p* ≤ 0.05, ^##^ *p* ≤ 0.01, ^###^ *p* ≤ 0.001.

We next tested whether spirodiclofen or zoxamide induced adipogenesis in mouse bone marrow-derived mesenchymal stem cells (mBMSCs) and 3T3-L1 preadipocytes since most (but not all) PPARγ activators increase adipogenesis (Janesick and Blumberg 2011b). Triflumizole and triphenyltin are not reported here because we previously published the results obtained with triflumizole (Li et al. 2012) and triphenyltin is a known obesogen (Kanayama et al. 2005). In 3T3-L1 preadipocytes, spirodiclofen induced adipogenesis at all doses tested, and zoxamide induced adipogenesis at the lowest dose (Figure 2A). In mBMSCs, spirodiclofen induced adipogenesis at 10 and 20 μM, whereas zoxamide induced differentiation at 2 and 10 μM (Figure 2B). Zoxamide was toxic to 3T3-L1 cells at ≥ 10 μM and to mBMSCs at ≥ 20 μM. QPCR evaluated expression of genes known to be involved in different phases of adipogenic differentiation (*Fabp4* for preadipocytes, *Fsp27* for lipid droplet accumulation, and *Lpl* for terminal differentiation). Corresponding increases in adipogenic gene expression were observed for spirodiclofen and zoxamide (Figure 2A,B).

### Selection of Assays for ToxPi Construction

**Figure 3:**
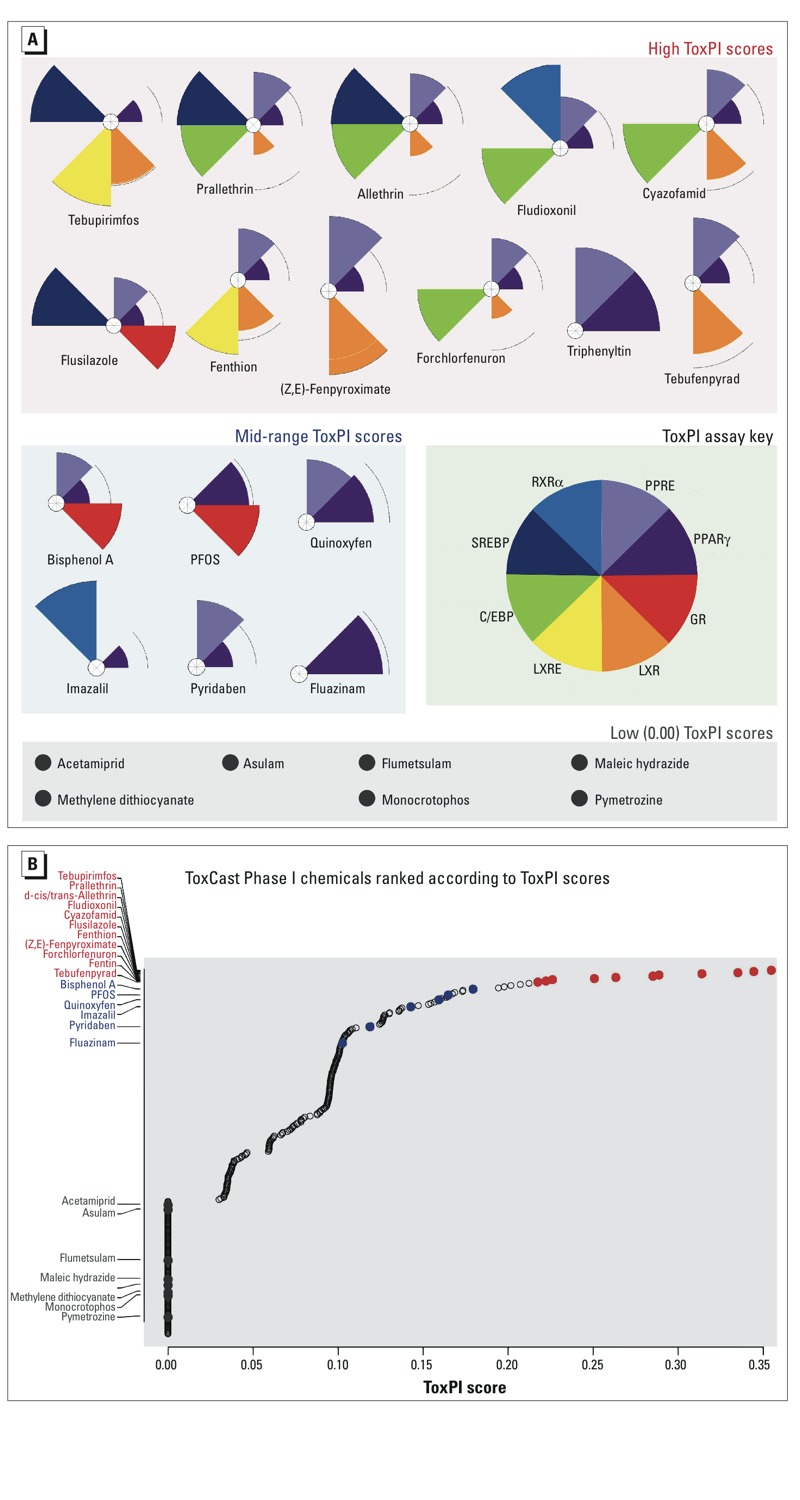
Selection of ToxPi Chemicals for Adipogenesis Assays. (*A*) Adipogenesis ToxPi where slice size (magnitude) represents the activity of a ToxCast^™^ chemical in a particular assay or collection of assays (see the assays that comprise each slice in Table S1 and the AC_50_ values associated with these assays in Table S3). PPRE, Attagene cis-PPRE reporter gene assay; PPARγ, Attagene and NCGC trans-PPARγ reporter gene assay and NovaScreen^®^ hPPARγ direct binding assay; GR, Attagene cis-GRE, trans-GR, and NCGC trans-GR reporter gene assay, and NovaScreen^®^ hGR direct binding assay; LXR, Attagene trans-LXRα, trans-LXRβ and NCGC trans-LXRβ reporter gene assay; LXRE, Attagene cis-LXRE reporter gene assay; C/EBP, Attagene cis-C/EBP reporter gene assay; SREBP, Attagene cis-SREBP reporter gene assay; RXRα, Attagene and NCGC trans-RXRα reporter gene assay. Highest scoring ToxPi chemicals are predicted to be obesogenic. (*B*) Plot of the ToxPi scores for all Phase I ToxCast^™^ chemicals. Red data points are selected high-scoring chemicals. Blue data points are selected medium-scoring chemicals. Grey data points are selected zero-scoring chemicals. Black open circles are chemicals not tested in our adipogenesis assays. PFOS, perfluorooctanesulfonic acid.

ToxPi is a prioritization tool that combines information from several assays to link chemicals with a particular biological process (Reif et al. 2010). Each ToxPi slice represents one assay or a collection of assays on the same target (see key, Figure 3A). Sixteen assays were chosen for the adipogenesis ToxPi, which were grouped into 8 slices (see Table S1). For example, the PPARγ slice consists of 3 assays because these assays were all performed on the same target, PPARγ, but executed by different companies with different methods (e.g., binding assay versus activation assay). The size of each ToxPi slice reflects the magnitude of the AC_50_ values (the lower the values, the larger the slice). PPARγ and RXRα were chosen for their ability to regulate fat cell development (Grün and Blumberg 2006; Rosen and MacDougald 2006; Tontonoz and Spiegelman 2008). Proteins of the C/EBP family function downstream and upstream of PPARγ to stabilize the adipogenic fate (Darlington et al. 1998; Rosen and MacDougald 2006). The glucocorticoid receptor (GR) and sterol regulatory element-binding protein (SREBP) both regulate lipid metabolism (Peckett et al. 2011; Raghow et al. 2008). LXR is responsible for adipocyte function and regulates SREBP-1c expression (Calkin and Tontonoz 2012).

**Figure 4:**
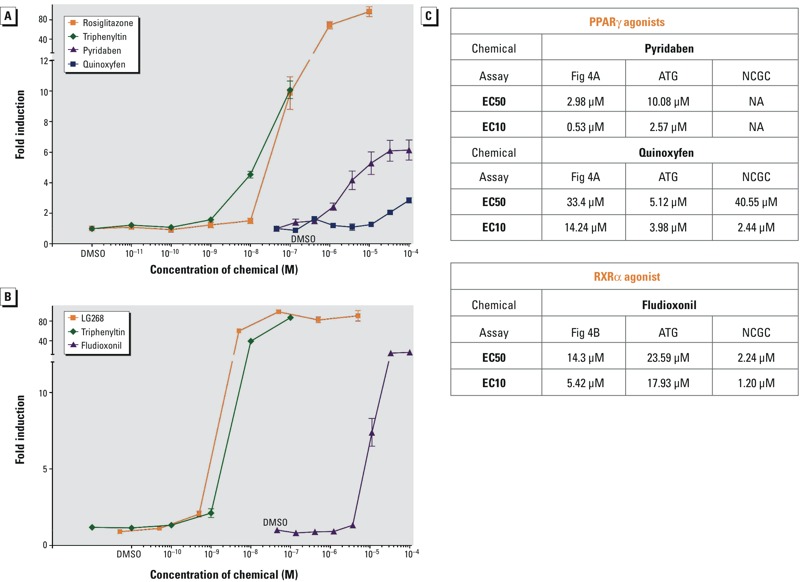
ToxPi Chemical Activity on PPARγ and RXRα. The ability of a graded dose series of ToxPi chemicals to activate (*A*) GAL4-mPPARγ or (*B*) GAL4-hRXRα was tested in transiently transfected COS7 cells. (*A*, *B*) Data points are averages of triplicate transfections (three biological replicates). Cytotoxicity, as measured by decreased β-galactosidase activity was observed at 1 μM for triphenyltin. ToxPi chemicals were tested in 3-fold serial dilutions from 100 μM through 0.137 μM, with the final data point being 0.05% DMSO. Data are depicted as fold induction over vehicle (0.05% DMSO) controls ± SEM. (*A*) Rosiglitazone serves as a positive control activator for GAL4-mPPARγ. (*B*) LG100268 (2-[1-(3,5,5,8,8-Pentamethyl-5,6,7,8-tetrahydro-2-naphthyl)cyclopropyl]pyridine-5-carboxylic acid) serves as a positive control activator for GAL4-hRXRα. (*C*) EC_50_ and EC_10_ values calculated from *A* and *B* are reported and compared to other assays (see Figure S1). ATG, Attagene GAL-PPARγ or GAL-RXRα activation assay; NCGC, GeneBLAzer^®^ GAL-PPARγ or GAL-RXRα activation assays. Triphenyltin was previously published (Kanayama et al. 2005).

No chemical was found to be active in all 16 assays. The highest scoring chemicals were active in 5–6 assays or 4–5 slices. Medium scoring chemicals were active in 1–3 slices/assays and low-scoring chemicals were not active in any assays. The low-scoring “negatives” also did not demonstrate activity in any other ToxPis that represented collective assays on feeding behavior, islet cell function, and insulin sensitivity (K. Thayer, personal communication, 14 December 2012). Figure 3A and Table S3 show 24 top-, medium-, low- (zero/negative) scoring chemicals obtained by ToxPi analysis. Figure 3B shows how these 24 chemicals rank in context with all ToxCast™ Phase I chemicals. We tested these chemicals in PPARγ and RXRα activation assays and found that pyridaben, quinoxyfen, and triphenyltin activated PPARγ (Figure 4A) and fludioxonil activated RXRα (Figure 4B). This means that only 2 out of the 11 high-scoring ToxPi chemicals and 2 out of the 6 medium-scoring ToxPi chemicals could activate PPARγ or RXRα despite that Attagene assays reported all as PPARγ or RXRα activators. Triphenyltin, a known PPARγ and RXRα agonist, was not on the Attagene list of RXRα activators (false negative). As expected, none of the low-scoring (zero) chemicals activated PPARγ or RXRα.

### Effects of Phase I ToxPi Predicted Adipogenic Chemicals on Adipogenesis

**Figure 5:**
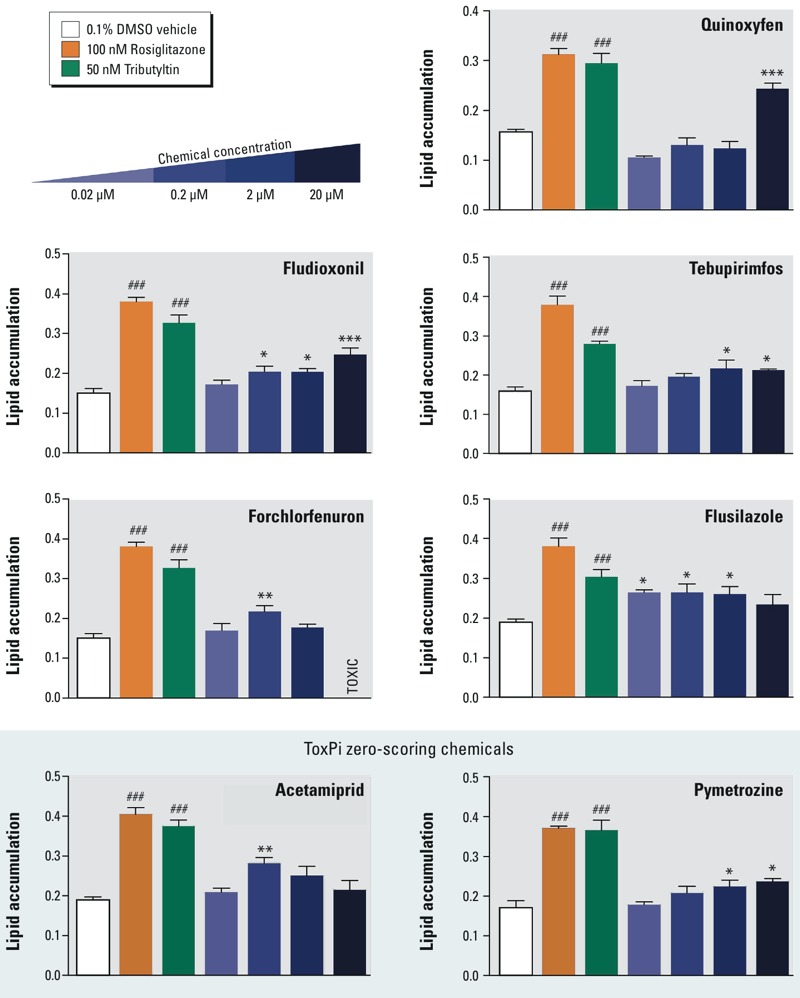
ToxPi chemicals induce adipogenesis in 3T3-L1 preadipocytes. Adipogenesis was induced in 3T3-L1 cells according to Figure S2. 3T3-L1 cells were exposed to adipogenic cocktail for 2 days, then exposed to the test chemicals for 5 days. Differentiated cells were fixed and stained with Nile red and Hoechst 33342. Lipid accumulation was quantified in cells by measuring Nile red fluorescence normalized by cell number (Hoechst). Rosiglitazone and tributyltin serve as positive control adipogenic chemicals. One-way ANOVA was conducted for ToxPi chemical treatment groups and DMSO vehicle, followed by Dunnett’s post hoc test: *p ≤ 0.05, **p ≤ 0.01, ***p ≤ 0.001 compared to vehicle. Unpaired *t*-test was conducted for the positive controls rosiglitazone, tributyltin versus vehicle: ^###^ *p* ≤ 0.001.

**Figure 6:**
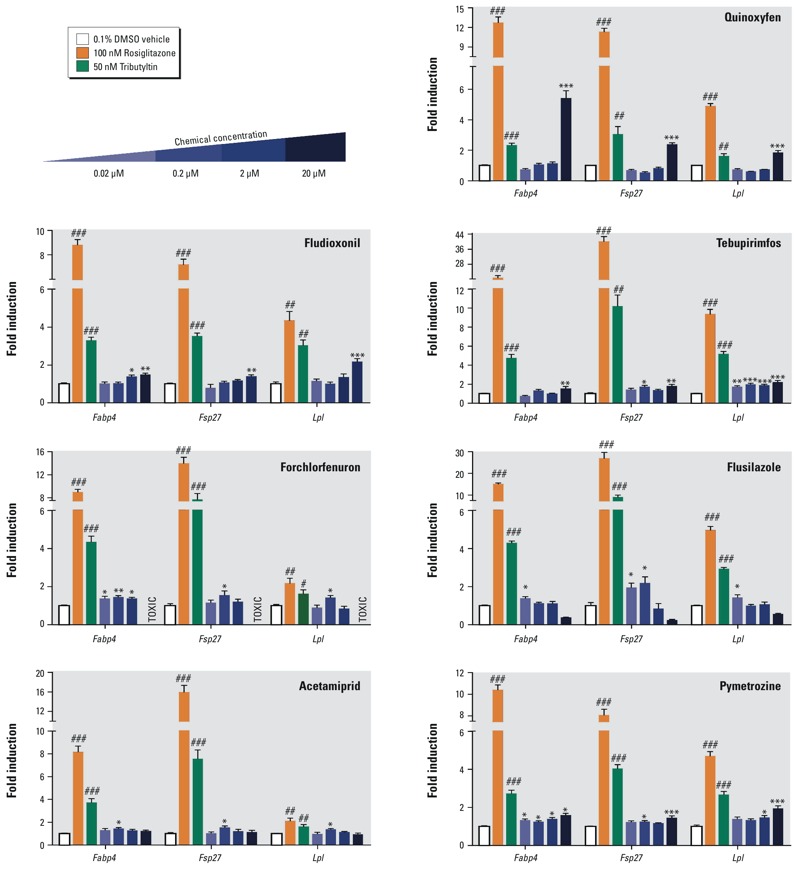
ToxPi chemicals induce adipogenic gene expression in 3T3-L1 preadipocytes. Adipogenesis was induced in 3T3-L1 cells according to Figure S2. 3T3-L1 cells were exposed to adipogenic cocktail for 2 days, then exposed to the test chemicals for 5 days. 3T3-L1 cells were homogenized in TriPure, total RNA was isolated, reverse transcribed, and QPCR was performed. Gene expression was determined by the 2^–^^ΔΔ^ ^CT^ method using* 36b4* as the reference gene. Data are reported as fold induction over 0.1% DMSO vehicle controls ± S.E.M using standard propagation of error. Primer sequences can be found in Table S4. One-way ANOVA was conducted for ToxPi treatment groups and DMSO vehicle, followed by Dunnett’s post hoc test: *p ≤ 0.05, **p ≤ 0.01, ***p ≤ 0.001 compared to vehicle. Unpaired *t*-test was conducted for the positive controls ROSI, TBT versus vehicle: ^#^ *p* ≤ 0.05, ^##^ *p* ≤ 0.01, ^###^ *p* ≤ 0.001.

**Figure 7:**
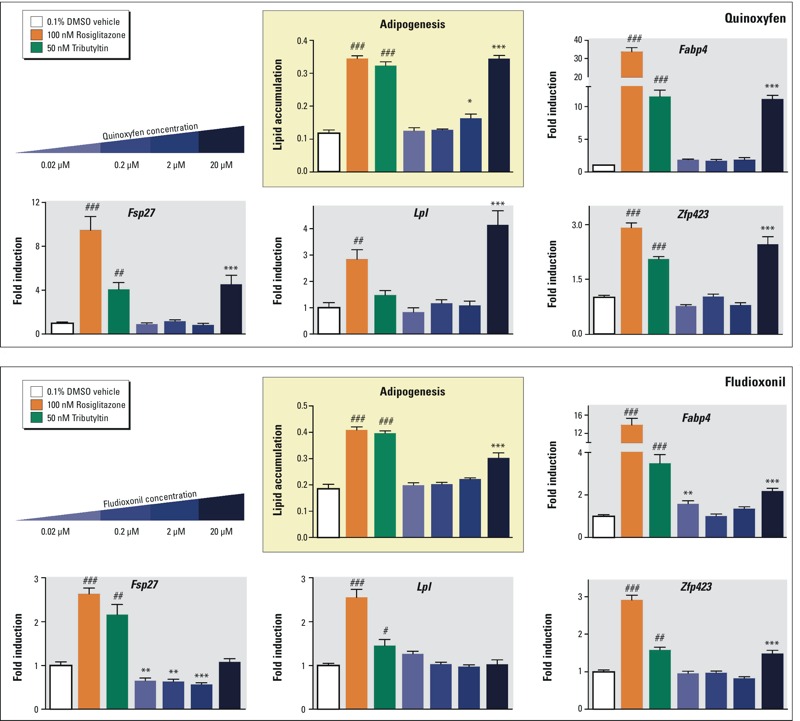
ToxPi chemicals quinoxyfen and fludioxonil induce adipogenesis in mBMSCs. Adipogenesis was induced in mouse bone marrow derived mesenchymal stem cells (mBMSCs) according to Figure S2. mBMSCs were exposed to adipogenic cocktail plus test chemicals or positive controls for 14 days. Differentiated cells were fixed and stained with Nile red and Hoechst 33342. Lipid accumulation was quantified in differentiated cells by measuring Nile red fluorescence normalized by cell number (Hoechst). Rosiglitazone (ROSI) and tributyltin (TBT) serve as positive control adipogenic chemicals. mBMSCs were homogenized in TriPure, total RNA was isolated, reverse transcribed, and QPCR was performed. Gene expression was determined by the 2^–^^ΔΔ^ ^CT^ method using *36b4* as the reference gene. Data are reported as fold induction over 0.1% DMSO vehicle controls ± S.E.M using standard propagation of error. Primer sequences can be found in Table S4. One-way ANOVA was conducted for ToxPi chemical treatment groups and DMSO vehicle, followed by Dunnett’s post hoc test: *p ≤ 0.05, **p ≤ 0.01, ***p ≤ 0.001 compared to vehicle. Unpaired *t*-test was conducted for the positive controls rosiglitazone, tributyltin versus vehicle: ^#^ *p* ≤ 0.05, ^##^ *p* ≤ 0.01, ^###^ *p *≤ 0.001.

The main goal of the adipogenesis ToxPi was not to assess individual receptor or transcription factor activators, but rather, to predict which chemicals might activate one or more key pathways that collectively promote adipogenesis. We tested all of the top-, medium-, and low- (zero/negative) chemicals obtained by ToxPi analysis in the 3T3-L1 preadipocyte model (Figure 5). We did not test triphenyltin, triflumizole, or bisphenol A here, because these have been previously published (Chamorro-García et al. 2012; Kanayama et al. 2005; Li et al. 2012; Masuno et al. 2002). Counting these known obesogens, 7 of the 17 of the top- and medium-scoring chemicals demonstrated adipogenic activity. Two out of seven of the negative, zero-scoring chemicals, acetamiprid and pymetrozine, promoted adipogenesis. Pyridaben strongly inhibited adipogenesis, despite its ability to activate PPARγ (see Figure S3). Figure 5 shows the results of an example 3T3-L1 assay, performed in triplicate and corresponding gene expression in Figure 6. QPCR in Figure 6 evaluated expression of genes known to be involved in different phases of adipogenic differentiation (*Zfp423* for early commitment, *Fabp4* for preadipocytes, and *Lpl* for terminal differentiation). These assays were repeated multiple times by a succession of laboratory personnel and showed similar results (data not shown). All chemicals that were positive in 3T3-L1 assays were tested in mBMSCs to evaluate which chemicals can promote differentiation of mesenchymal stem cells (MSCs)into adipocytes. We identified fludioxonil and quinoxyfen as obesogenic chemicals that could differentiate these uncommitted stem cells into adipocytes (Figure 7), whereas the others could only induce differentiation in cells already committed to the adipocyte lineage (preadipocytes). Gene expression analysis by QPCR verified the *in vitro* cell culture results (Figure 7).

### ToxCast™/NCGC Discrepancies and Summary of Phase I ToxCast™ and ToxPi Data

**Figure 8:**
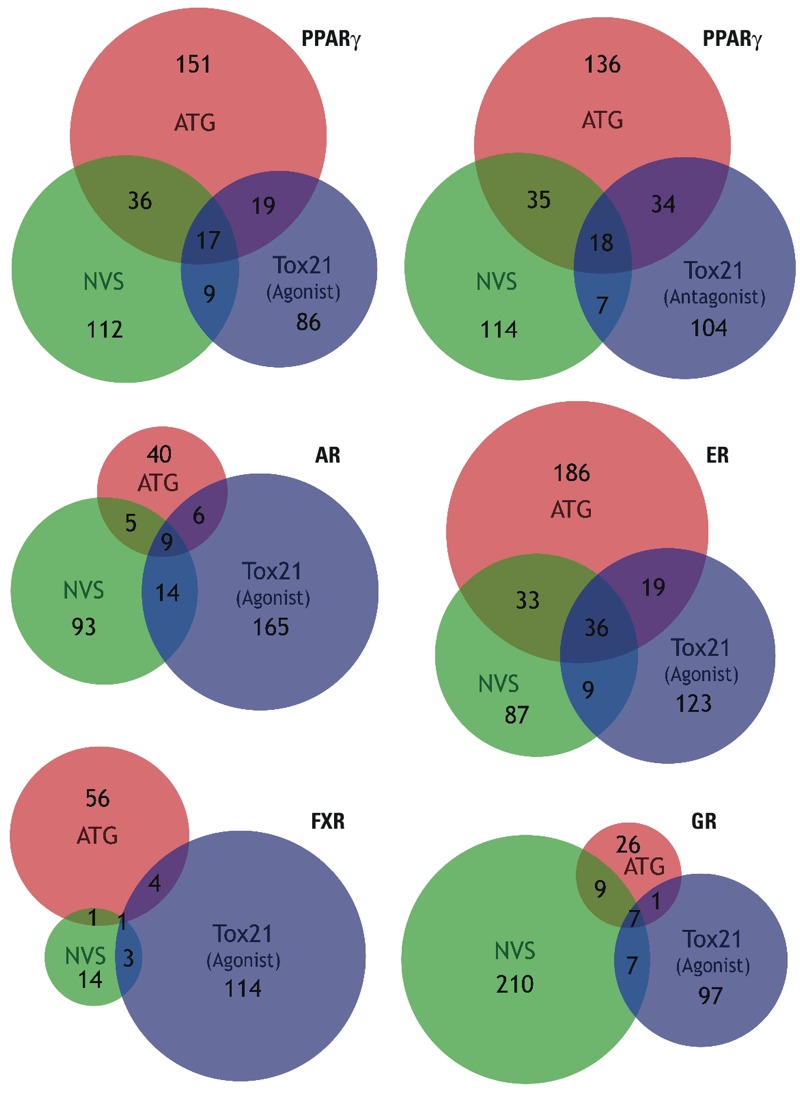
Venn diagrams comparing three main nuclear receptor commercial assays employed in ToxCast™. Phase II, release 2014 (Filer et al. 2014) assay datasets (gain AC_50_ values) were obtained for five nuclear receptors: PPARγ, androgen receptor (AR), estrogen receptor (ER), farnesoid X receptor (FXR), and glucocorticoid receptor (GR). Three assays for each receptor were evaluated: Attagene (ATG) agonist assay (red), NovaScreen^®^ (NVS) direct binding assay (green), and NCGC/Tox21 GeneBLAzer^®^ agonist assay (blue). An additional diagram (top right) was created for PPARγ using NCGC/Tox21 antagonism assay. Chemicals scoring AC_50_ ≤ 10 μM for each assay were incorporated in the Venn diagrams, which were created by BioVenn (Hulsen et al. 2008).

We created summary tables that describe all chemicals we tested and compare the various activation and adipogenic assays available. Table 1 is a summary of the ToxCast™, PPARγ activation analysis and Table 2 is a summary of the ToxPi analysis. Tables S6 and S7 are a continuation of these tables and show a comparison of AC_50_ values from our assays, Attagene, NovaScreen®, and NCGC/Tox21. Notable discrepancies between the assay platforms on the same receptor end point are apparent. The most fundamental problem is that the three main nuclear receptor assays—Attagene, NovaScreen®, and NCGC/Tox21—do not overlap nearly as well as would be expected, even using the most current Phase II ToxCast™ data release (Filer et al. 2014) (Figure 8). The Attagene PPARγ agonist assay has proportionally more overlap with the NCGC/Tox21 PPARγ *antagonist*, rather than the agonist assay. When taking the intersection of Phase II, 2014 release data for Attagene, NovaScreen®, and NCGC/Tox21 agonist assays, we found 17 chemicals, and even these are unlikely to be true activators. For example, docusate sodium is a detergent and, while it was recently shown to activate PPARγ (Temkin et al. 2016), it is also likely to be a pan-assay interference compound (Dahlin et al. 2015) because it is positive in 214 ToxCast™ assays. Fluazinam is a PPARγ antagonist (Figure 1) that did not promote adipogenesis, and tetrac is a thyroid hormone analog, unlikely to have affinity for PPARγ. Moreover, Attagene identifies 100 chemicals that activate RXRβ but not RXRα (see Figure S4), despite that receptor selective rexinoids are not known to exist because the same residues make contact with ligand in all three RXR subtypes: RXRα, RXRβ, and RXRγ (Love et al. 2002). These results are *prima facie* implausible and should have indicated to the screeners that one or more of the assays are problematic.

**Table 1:** Summary of results from Figures 1 and 2.

ToxCast^™^ chemical^*a*^ Chemical name	Adipogenesis^*b*^	Activation^*c*^	
	3T3-L1	COS7	AC_50_
Triphenyltin^*d*^	Positive	PPARγ activator	0.02
Fluazinam^*d*^	Negative	PPARγ antagonist	7.2
Niclosamide	Not tested	Inactive
Pyraclostrobin	Not tested	Inactive
Zoxamide	Positive	PPARγ activator	1.31
Acetochlor	Negative	PPARγ antagonist	46.7
Butachlor	Not tested	Not tested
Triflumizole	Positive	PPARγ activator	11.5
Prochloraz	Not tested	Inactive
Spirodiclofen	Positive	PPARγ activator	12.76
Alachlor	Negative	PPARγ antagonist	4.48
Tebufenpyrad^*d*^	Not tested	Inactive
Dimethenamid	Not tested	Inactive
Tebufenozide	Not tested	Inactive
Quinoxyfen^*e*^	Not tested	Inactive
Indoxacarb	Not tested	Inactive
Fenpyroximate (Z,E)^*d*^	Not tested	Inactive
S-Bioallethrin	Not tested	Inactive
Dimethomorph	Not tested	Inactive
Cyazofamid^*d*^	Not tested	Inactive
Chlorothalonil	Not tested	Inactive
^***a***^List of the chemicals used in PPARγ activation or antagonism assays (Figure 1). ^***b***^Results of the 3T3-L1 adipogenesis assay. Only those chemicals that were positive activators on PPARγ were tested, and all those tested were adipogenic. ^***c***^Results of the Cos-7 transient transfection assays, with the AC_50_ value (in μM) listed. Table S6 has a continuation of this table where the AC_50_ values are compared to commercial assays. ^***d***^Chemicals that were also tested in ToxPi assays (Table 2). ^***e***^Quinoxyfen was later shown to be active at 100 μM (Table 2).			

**Table 2:** Summary of ToxPi results derived from Figures 3–7.

ToxPi chemical^*a*^ Chemical name	Prioritization^*b*^	Adipogenesis^*c*^	Activation^*d*^	
	ToxPi score	3T3-L1	COS7	AC_50_
Tebupirimfos	HIGH	Positive	Inactive	
Prallethrin	HIGH	Negative	Inactive	
d-cis/trans Allethrin	HIGH	Negative	Inactive	
Fludioxonil^*f*^	HIGH	Positive	RXRα activator	14.3
Cyazofamid^*e*^	HIGH	Negative	Inactive	
Flusilazole	HIGH	Positive	Inactive	
Fenthion	HIGH	Negative	Inactive	
Fenpyroximate (Z,E)^*e*^	HIGH	Negative	Inactive	
Forchlorfenuron	HIGH	Positive	Inactive	
Triphenyltin^*e*^	HIGH	Positive	PPARγ activator	0.02
Tebufenpyrad^*e*^	HIGH	Negative	Inactive
Bisphenol A	MEDIUM	Positive	Inactive
PFOS	MEDIUM	Negative	Inactive
Quinoxyfen^*e*^^,^^*f*^	MEDIUM	Positive	PPARγ activator	33.4
Imazalil	MEDIUM	Negative	Inactive	
Pyridaben	MEDIUM	Inhibitor	PPARγ activator	3.0
Fluazinam^*e*^	MEDIUM	Negative	PPARγ antagonist	7.2
Methylene dithiocyanate	NEGATIVE	Negative	Inactive
Maleic hydrazide	NEGATIVE	Negative	Inactive
Monocrotophos	NEGATIVE	Negative	Inactive
Asulam	NEGATIVE	Negative	Inactive
Flumetsulam	NEGATIVE	Negative	Inactive
Acetamiprid	NEGATIVE	Positive	Inactive
Pymetrozine	NEGATIVE	Positive	Inactive
^***a***^List of the chemicals used in PPARγ or RXRα activation assays and adipogenesis assays (Figures 4–7). ^***b***^ToxPi score (Reif et al. 2010, 2013) (Figure 3). ^***c***^Results of the 3T3-L1 adipogenesis assay. ^***d***^Cos-7 transient transfection assays, with the AC_50_ value (in μM) listed. Table S7 has a continuation of this table where the AC_50_ values are compared to commercial assays. ^***e***^Chemicals that were also tested in ToxCast^™^ assays (Table 1). ^***f***^These chemicals promoted adipogenesis in mBMSCs in addition to 3T3-L1 cells.				

*Z-*score corrections are currently being implemented by ToxCast™ as a mechanism to remove false positive chemicals. *Z-*scores are a measurement of potency relative to cytotoxicity of each chemical-assay pair (U.S. EPA 2014a). Typically, a chemical with a *Z-*score less than 3 is either removed, or flagged as a “non-selective” hit (U.S. EPA 2014a). Recently, Phase II ToxPi diagrams have been constructed by first removing chemicals with low *Z-*scores, and then correcting the magnitude of the Pi slice by adding the *Z-*score to the log(AC_50_) (Kris Thayer, personal communication). We have used Phase II, 2014 release data (Filer et al. 2014) to regenerate a list of PPARγ activators and adipogenesis ToxPi employing *Z-*score corrections for the Phase I chemical library. On the positive side, applying *Z-*scores, nearly all false positive ToxPi chemicals are lost, or ranked very low. Incredibly, all true positives we identified are also lost (see Figure S5A). (Z,E)-Fenproximate continues to be ranked high in all analyses, but we showed that this chemical was not adipogenic. Acetamiprid and Pymetrozine, which we found to be adipogenic, were false negatives in ToxPi Phase I 2011 release (Knudsen et al. 2011) and their ranking does improve slightly in 2014 release (Filer et al. 2014), especially with *Z-*scores incorporated. Top scoring chemicals from the ToxPi using Phase I chemical library but Phase II data are shown with (see Figure S6A) and without (see Figure S6B) *Z-*score correction. Pyridaben appears in the high ranking, non-*Z-*score corrected hits, yet, we found that this chemical inhibited adipogenesis (see Figure S3).

When investigating the PPARγ assays only, we found that Phase II data using *Z-*score correction identifies an almost entirely new set of top-scoring chemicals (see Table S5). As we observed with the Phase II ToxPi data, *Z-*scores can alter results dramatically (see Figure S5B). Nine out of 12 false positive chemicals are removed, while 3 out of 12 (tebufenpyrad, pyraclostrobin, and dimethenamid) remain. Unfortunately, the bona fide PPARγ activators, quinoxyfen and triflumizole, are also eliminated by their *Z-*scores, and triphenyltin is an inactive chemical in all three PPARγ assays. Zoxamide is only called active in 1 out of 3 assays and has a relatively small *Z-*score, and therefore is ranked quite low. Instead, chemicals such as atrazine and 2,4-D (2,4-dichlorophenoxyacetic acid) are ranked higher. Since these chemicals were in hand, we tested them on PPARγ and RXRα and found them to be inactive (see Figure S7). We created a second table without *Z-*score correction and found that the list of chemicals also differed from the original list (see Table S5). This is primarily due to the poor correlation between assay results in the Phase I 2011 release (Knudsen et al. 2011) versus the Phase II 2014 release (Filer et al. 2014) (see Figure S8). We tested the top-scoring chemical, triclosan, and found it to be inactive on PPARγ (see Figure S7). Taken together, these data suggest that recent refinements made to the analyses of ToxCast™ data alone do not improve their ability to measure PPARγ or RXR activity or to predict adipogenic capacity.

## Discussion

The ToxCast™ program is a high-throughput screening effort initiated by the U.S. EPA to predict chemical toxicity and for potential use in risk assessment. More recently, ToxCast™ has been repurposed as a prescreening effort to identify chemicals that should be screened using the full battery of tests in the EDSP. This approach shows some promise for identifying chemicals that disrupt androgen and estrogen receptors (Rotroff et al. 2013, 2014). We evaluated the ability of ToxCast™ assays to predict obesogenic chemicals by measuring their ability to activate PPARγ and RXRα in transient transfection assays and to promote adipogenesis in MSCs and 3T3-L1 preadipocytes. Our results show that ToxCast™ assays were able to successfully predict some bona fide obesogens; however, this success was accompanied by numerous false positives and a few false negatives. In our first study, we worked with a list of 21 chemicals reported to be PPARγ activators in ToxCast™ Phase 1 assays. We could only validate 5 out of 21 of these chemicals as PPARγ activators: triphenyltin, zoxamide, triflumizole, spirodiclofen, and quinoxyfen. Moreover, 3 of 21 (fluazinam, acetochlor, and alachlor) were weak PPARγ antagonists. On the positive side, each of the bona fide PPARγ activators promoted adipogenesis in 3T3-L1 cells and mBMSCs. This finding suggests that well-executed PPARγ-activation assays could be informative.

In our second study, we identified a set of gene targets whose regulation could be relevant to adipogenesis and used ToxCast™ Phase 1 assays and ToxPi software to generate a list of candidate chemicals for testing. We found that 5 out of 11 high-scoring and 2 out of 6 medium-scoring ToxPi chemicals were adipogenic in 3T3-L1 preadipocytes. Surprisingly, 2 out of 7 of the predicted negatives were adipogenic. Of the 9 chemicals that could differentiate 3T3-L1 preadipocytes, only 3 [quinoxyfen, triflumizole (Li et al. 2012), and fludioxonil], were able to induce adipogenesis in uncommitted mBMSCs. Perhaps not surprisingly, these 3 chemicals were PPARγ (quinoxyfen, triflumizole) or RXR (fludioxonil) activators. One additional notable result was that the PPARγ activator pyridaben was a strong inhibitor of adipogenesis. While uncommon, this result is not unprecedented; mycophenolic acid is a known PPARγ activator that inhibits adipogenesis (Ubukata et al. 2007). There are two possible conclusions for the poor predictive power of the adipogenesis ToxPi. The first is that we did not identify an appropriate group of assays and that this resulted in the poor performance of the ToxPi. The second possibility is that the very poor correlation between receptor activation reported in ToxCast™ assays and bona fide receptor activation we measured resulted in poor predictive power of the adipogenesis ToxPi.

It is interesting to consider why the current ToxCast™ strategy is relatively effective for the androgen and estrogen receptors (Rotroff et al. 2014) but performs poorly for predicting PPARγ activity or obesogenicity. One possibility is that PPARγ has a large binding pocket with relatively few high-affinity endogenous ligands, whereas ER and AR bind endogenous ligands at subnanomolar levels. For this reason, many PPARγ activators might be identified by ToxCast™ without being biologically relevant. Another possibility is that the much larger number of ER and AR assays in ToxCast™ limits the damage caused by a few poorly performing assays to the predictive power of the overall assay system. However, false positive problems were also recently observed in ToxCast™ AR and ER endocrine-disruption and neurotoxicity assays (Silva et al. 2015). Another possibility is that Phase I data only considered AC_50_ values. Currently, in ToxCast™ Phase II, a more sophisticated approach has been implemented that incorporates measures of cytotoxicity and chemicals are assigned a so-called *Z-*score. It is recommended that chemicals with *Z-*scores < 3 should be removed, or at a minimum, flagged or filtered (U.S. EPA 2014a). When we apply these cytotoxicity measures to the ToxCast™ Phase I chemicals, most false positives are removed; however, all true positives are also lost. A new collection of PPARγ activators or adipogenic chemicals can be identified using *Z-*score corrections, but our data suggest that this new approach alone does not improve the ability of ToxPi models to predict adipogenic activity or PPARγ activators.

Although we identified new obesogens from the ToxCast™ Phase 1 dataset, the low validation rate of PPARγ and RXRα activation assays, coupled with the poor predictive power of the adipogenesis ToxPi is troublesome and prevents these data from being used as effective predictors of adipogenic activity. It was recognized by early computer programmers that the quality of the output from any software is dependent on the quality of the input: Reliable models cannot be produced from inaccurate data or results that cannot be reproduced. It is a *sine qua non* of high-throughput screening that HTS assays alone cannot accurately predict the activity of chemicals against any end point. In their seminal paper on high-throughput screening assays, Inglese et al. (2007) note that “It is essential to view the primary HTS as the initial step of an integrated process.” All HTS assay results need to be confirmed by counter-screens and secondary screens that reduce the number of false positives and false negatives (Hughes et al. 2012). To the best of our knowledge, ToxCast™ assays as currently practiced are not constructed in this manner and this limits their accuracy. Until the HTS assays reflect actual receptor activity, these data must be interpreted with caution. This is particularly important now that the U.S. EPA is proposing to use ToxCast™ as a substitute for EDSP Tier 1 assays (Browne et al. 2015).

Another issue is that the assays used in ToxCast™ were largely pre-existing commercial assays which were adopted from the philosophy and approach of the pharmaceutical industry. Assays for drug discovery are designed to identify only the strongest hits in large libraries of structurally similar chemicals (millions or more) to limit the subsequent screening required to develop lead compounds for preclinical studies. This is philosophically the opposite of a proper chemical genomics approach to identify potential bad actors that should be selected for further scrutiny. Such assays would seek to identify every chemical that activates a particular pathway in a statistically significant way and then rank these for further testing. The ability of ToxCast™ assays to predict *in vivo* toxicity is often evaluated by comparing the effects of a chemical in ToxCast™ with effects from guideline studies, *in vivo* (Rotroff et al. 2013). However, the end points in guideline studies are not always sensitive to chemical effects on the endocrine system (Zoeller et al. 2012); thus, limiting their utility as validators of ToxCast™ assays for endocrine activity.

Of particular concern with respect to ToxCast™ is the lack of agreement among assays on the same endpoint. Exacerbating this problem is that Attagene assays identified approximately two to three times more chemicals than other assays on both PPARγ and ER end points (Figure 8; also see Figure 6 in the supplemental material in Rotroff et al. 2014). Attagene trans-Factorial™ assays and NCGC GeneBLAzer^®^ assays utilize well-understood and thoroughly tested GAL4 DNA-binding domain, nuclear receptor ligand-binding domain chimeras that perform well in a laboratory setting. Therefore, an open and important question is why such well-established principles of nuclear receptor biology have been implemented so poorly, yet are relied upon without further validation. Furthermore, the suitability of Attagene cis-Factorial™ assays to identify effects on specific gene-regulatory pathways is fundamentally questionable due to obvious off-target effects. For example, the PPAR response element used in the Attagene cis-PPRE reporter gene assay will bind PPARδ and PPARα, RXRα,β,γ, COUP-TFα,β, and HNF4 (Nakshatri and Bhat-Nakshatri 1998) at a minimum. Therefore, the results of such an assay cannot reasonably be called PPAR-specific—leaving one to wonder why such assertions are accepted uncritically. Despite these weaknesses, ToxCast™ publications continually average results across all similar assays equally to create composite curves (Rotroff et al. 2014). The lack of correspondence between distinct, yet mechanistically similar or identical assays is ignored, and all assays are weighted equally (Rotroff et al. 2014). This may work for cases such as the estrogen receptor where the large number of assays reduces the negative impact of the poorly performing assays but will necessarily fail when assay numbers are small (such as for PPARγ, RXR, etc.). It would be beneficial for other measures of action on particular end points to be included.

## Conclusions

We have identified several problems in the ToxCast™ Phase I and Phase II data that impair the ability of these assays to predict activity on PPARγ, RXR and adipogenesis. We recommend eliminating the practice of averaging results across assays in favor of eliminating poorly performing assays. We recommend incorporating reliable counter-screens and secondary screens to validate the results of primary HTS assays before these are used for prioritization of chemical lists for further testing or to inform regulatory testing. Such modifications would be very beneficial and could improve the performance of ToxCast™ such that it can be as useful as originally envisioned. It is time that ToxCast™ assays and approaches are modified so that they produce accurate results that can be validated in subsequent experiments by multiple laboratories at high frequency.

## Supplemental Material

(3.7 MB) PDF
