# Supplementary material for: On the Utility of ToxCast™ and ToxPi as Methods for Identifying New Obesogens
Source: Environ Health Perspect. 2016 Jan 13;124(8):1214–26. doi: 10.1289/ehp.1510352 (PMC4977052; doi:10.1289/ehp.1510352)
Supplement: (3.7 MB) PDF [file ehp.1510352.s001.acco.pdf]

Environ Health Perspect
DOI: 10.1289/ehp.1510352

Note to readers with disabilities: *EHP* strives to ensure that all journal content is accessible to all readers. However, some figures and Supplemental Material published in *EHP* articles may not conform to 508 standards due to the complexity of the information being presented. If you need assistance accessing journal content, please contact ehp508@niehs.nih.gov. Our staff will work with you to assess and meet your accessibility needs within 3 working days.

Supplemental Material

On the Utility of ToxCast™ and ToxPi as Methods for Identifying New Obesogens

Amanda Shaine Janesick, Giorgio Dimastrogiovanni, Lenka Vanek, Christy Boulos, Raquel Chamorro-García, Weiyi Tang, and Bruce Blumberg

Table of Contents

Figure S1. Regression analysis of activation and antagonism curves from Figures 1 and 4. Data points are averages of triplicate transfections (3 biological replicates). Data are depicted as fold induction over vehicle (0.05% DMSO) controls ± S.E.M. EC_{50} and IC_{50} values were obtained using nonlinear regression, variable slope in GraphPad Prism 5.0 Spirodiclofen did not plateau, therefore, it was constrained at the top dose.

Figure S2. Schematic of adipogenesis assays. Top. 3T3-L1 cells were seeded at $2x10^4$ cells per well in 12-well plates. After 48 hours, cells were exposed to the adipogenic cocktail MDI (isobutyl-methylxanthine, dexamethasone, and insulin) for 2 days. Induction media was removed and cells were exposed to ToxCast chemicals (or controls) during 5 days replacing the media every 2 days. **Bottom.** $8x10^4$ cells/well mBMSCs were maintained as subconfluent monolayers in basic medium. Once confluent, mBMSCs were induced to differentiate with adipogenic cocktail and ToxCast chemicals (or controls) during 14 days replacing the media every 3 days.

Figure S3. ToxPi chemical pyridaben inhibits adipogenesis in 3T3-L1 preadipocytes. Adipogenesis was induced in cells according to Supplemental Material, Figure S2. Lipid accumulation was assessed by measuring the percent of surface area in each well covered by Oil Red 0 positive cells using Image J software. One-way ANOVA was conducted for

pyridaben treatment groups and DMSO vehicle (VEH), followed by Dunnett's post-hoc test: *** $P \leq 0.001$ compared to DMSO. Unpaired t-test was conducted for the positive control Rosiglitazone (ROSI) versus DMSO vehicle: ### $P \leq 0.001$.

Figure S4. Attagene identifies a suspiciously large number of RXR-selective chemicals. Phase II, release 2014 (Filer et al. 2014) datasets (gain AC_{50} values) were obtained for Attagene agonist assays on RXRα and RXRβ. Chemicals scoring $AC_{50} \leq 10$ µM for each assay were incorporated in the Venn diagrams, created by BioVenn (Hulsen et al. 2008). The Venn diagram reveals 100 RXRβ-selective and 30 RXRα-selective chemicals, with only 22 chemicals activating both receptor subtypes.

Figure S5. True positives and false positives are lost when employing ToxCast Phase II new models. (**A**) Green triangles: ToxPi Phase I rankings (based solely on AC_{50} values). Blue dots: ToxPi Phase II rankings using the Phase I chemical library are based solely on AC_{50} values. Squares: ToxPi Phase II rankings using Phase I chemical library were constructed by first removing chemicals with low Z-scores, and then correcting the magnitude each Pi slice by adding the Z-score to the negative log (AC_{50}). Differences between triangles and dots are due to discrepancies between Phase I and Phase II assays on the same chemical. Differences between squares and dots are attributed to the Z-score correction. (**B**) **Circles/dots:** Raw Z-score values derived from ToxPi Phase II data using Phase I chemical library. **Triangles:** Z-score corrected AC_{50} values derived from ToxPi Phase II data using Phase I chemical library. All true PPARγ activators rank significantly lower in both raw Z-score and Z-score corrected AC_{50} values compared to atrazine and quinclorac. However, atrazine is not a PPARγ activator in Cos7 transient transfection assays (Supplemental Material, Figure S7).

Figure S6. ToxPi regeneration using Phase II, 2014 dataset and Phase I chemical library. Adipogenesis ToxPi diagrams where slice size (magnitude) represents the activity of a ToxCast chemical in a particular assay or collection of assays (Supplemental Material, Table S1). Highest scoring ToxPi chemicals are predicted to be obesogenic. (**A**) Top scoring Phase II ToxPi chemicals with Z-score correction. The magnitude of the Pi slice is determined by adding the Z-score to the negative log (AC_{50}). (**B**) Top scoring Phase II ToxPi chemicals without Z-score correction, based solely on AC_{50} values.

Figure S7. Phase II ToxCast chemical activity on PPARγ and RXRα. The abil ty of a graded dose series of ToxCast chemicals to activate **(A)** GAL4-mPPARγ or (**B**) GAL4-hRXRα was tested in transiently transfected Cos7 cells. (A, B) Data points are averages of triplicate

transfections (3 biological replicates). Cytotoxicity, as measured by decreased β-galactosidase activity was observed at 33 μM and 100 μM for triclosan. Data are depicted as fold induction over vehicle (0.05% DMSO) controls ± S.E.M. (A) ToxCast chemicals were tested in 3-fold serial dilutions from 100 μM through 0.137 μM, with the final data point being 0.05% DMSO. Rosiglitazone (A) and AGN1934204 (B) serve as a positive control activators and were tested in 10-fold serial dilutions.

Figure S8. Linear regression analysis of ToxCast Phase I and Phase II assays on the same endpoint (same chemical/assay pair).

Table S1. 16 assays were used to construct the adipogenic ToxPi models shown in Figure 3. 16 assays from Attagene, NovaScreen, and NCGC were incorporated into ToxPi models (Figure 3, Supplemental Material, Table S3). These 16 assays were chosen because they were relevant to the biological process of adipogenesis. All information provided in this table is derived from ToxCast_Phase_1_Assays_20110110.txt at http://epa.gov/ncct/toxcast/data_archive.html (2011 release; Knudsen et al. 2011). ATG_RXRα_TRANS, ATG_GR_TRANS, ATG_GRE_CIS, and NCGC_GR_Agonist assays showed no activation by any of the 320 ToxCast chemicals. The leftmost column indicates the assay or collection of assays contributing to a particular slice. For example, the PPARγ slice in Figure 3A is comprised of three assays: ATG_PPARγ_TRANS, NCGC_PPARγ_Agonist, and NVS_NR_hPPARγ.

Table S2. List of top scoring chemicals in ToxCast PPARγ assays. AC_{50} values (μM) from 2 PPARγ agonist assays (Attagene-ATG, NCGC), 1 PPARγ direct binding assay (Novascreen-NVS), and 1 PPARγ antagonist assay (NCGC/Tox21) are shown here, ranked on the Attagene assay -- 2011 release (Knudsen et al. 2011). GSID = DSSTox chemical identifier; CASRN = CAS Registry Number; NA = not active.

Table S3. 24 top, medium, low (zero/negative) scoring chemicals obtained by ToxPi analysis. Data directly correspond to ToxPi diagrams shown in Figure 3. Assay descriptions are available in Supplemental Material, Table S1. AC_{50} values are expressed in μM. Inactive chemicals were not active in any of the ToxCast assays for adipogenesis, feeding behavior, islet cell function, or insulin sensitivity. ATGC = Attagene cis-Factorial™ assay; ATGT = Attagene trans-Factorial™ assay; NCGC = GeneBLAzer agonist assay; NVS = Novascreen direct binding assay; NA = not active.

Table S4. Primer sequences

Table S5. Re-evaluation of PPARγ activators using Phase II data for three ToxCast PPARγ assays. AC_{50} values (μM) and Z-scores from ToxCast 2014 release (Filer et al. 2014) is shown. (A) The Phase I chemical library was ranked based solely on AC_{50} values (μM) of the Attagene and Tox21/NCGC PPARγ assays and NovaScreen PPARγ direct binding assay. (B) Chemicals were first filtered by removing those with cytotoxicity Z-scores less than 3 (U.S. EPA 2014) and then ranking them based on their Z-score + negative log (AC_{50}). NA = not active.

Table S6. Continuation of Table 1: Comparison of results in Figures 1 and 2 versus ToxCast assay data. Column 1 is a list of chemicals used in our PPARγ activation/antagonism assays and adipogenesis assays. AC_{50} values (μM) from ToxCast 2011 (Knudsen et al. 2011) and 2014 (Filer et al. 2014) releases are shown. ATG = Attagene Factorial™ PPARγ agonist assay; NVS = NovaScreen PPARγ direct binding assay; NCGC = GeneBLAzer PPARγ agonist or antagonist assay; NA = not active.

Table S7. Continuation of Table 2: Comparison of results in Figures 3-7 versus ToxCast Phase I assay data. Column 1 is a list of the ToxPi chemicals (Figure 3) used in PPARγ or RXRα activation assays (Figure 4) and adipogenesis assays (Figures 5-7). AC_{50} values (μM) from ToxCast 2011 (Knudsen et al. 2011) and 2014 (Filer et al. 2014) releases are shown. ATG = Attagene Factorial™ agonist assay; NVS = NovaScreen direct binding assay; NCGC = GeneBLAzer agonist or antagonist assay; NA = not active.

References

Supplemental Material, Figure S1.

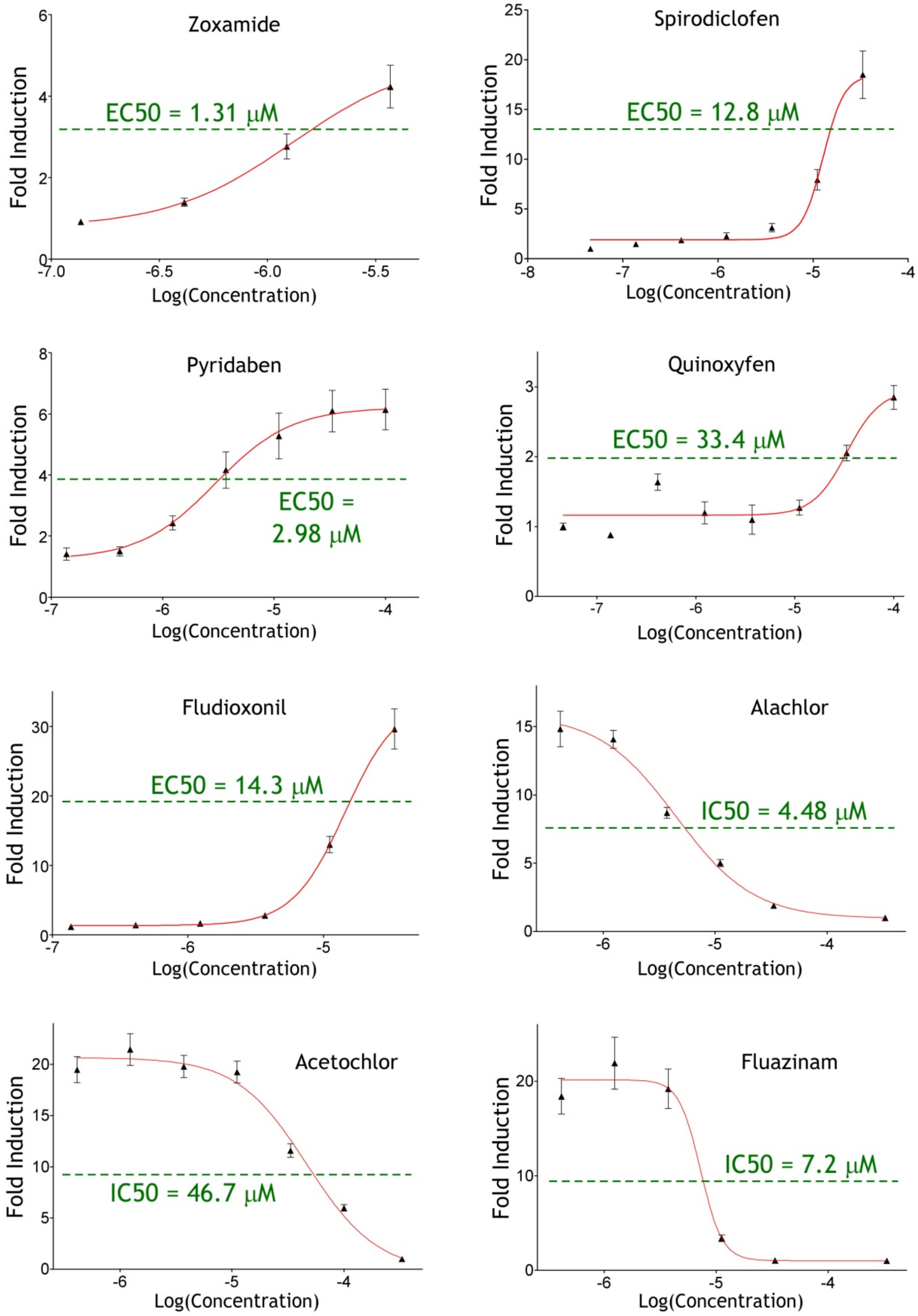

Supplemental Material, Figure S1. Regression analysis of activation and antagonism curves from Figures 1 and 4. Data points are averages of triplicate transfections (3 biological replicates). Data are depicted as fold induction over vehicle (0.05% DMSO) controls ± S.E.M. EC_{50} and IC_{50} values were obtained using nonlinear regression, variable slope in GraphPad Prism 5.0 Spirodiclofen did not plateau, therefore, it was constrained at the top dose.

Supplemental Material, Figure S2.

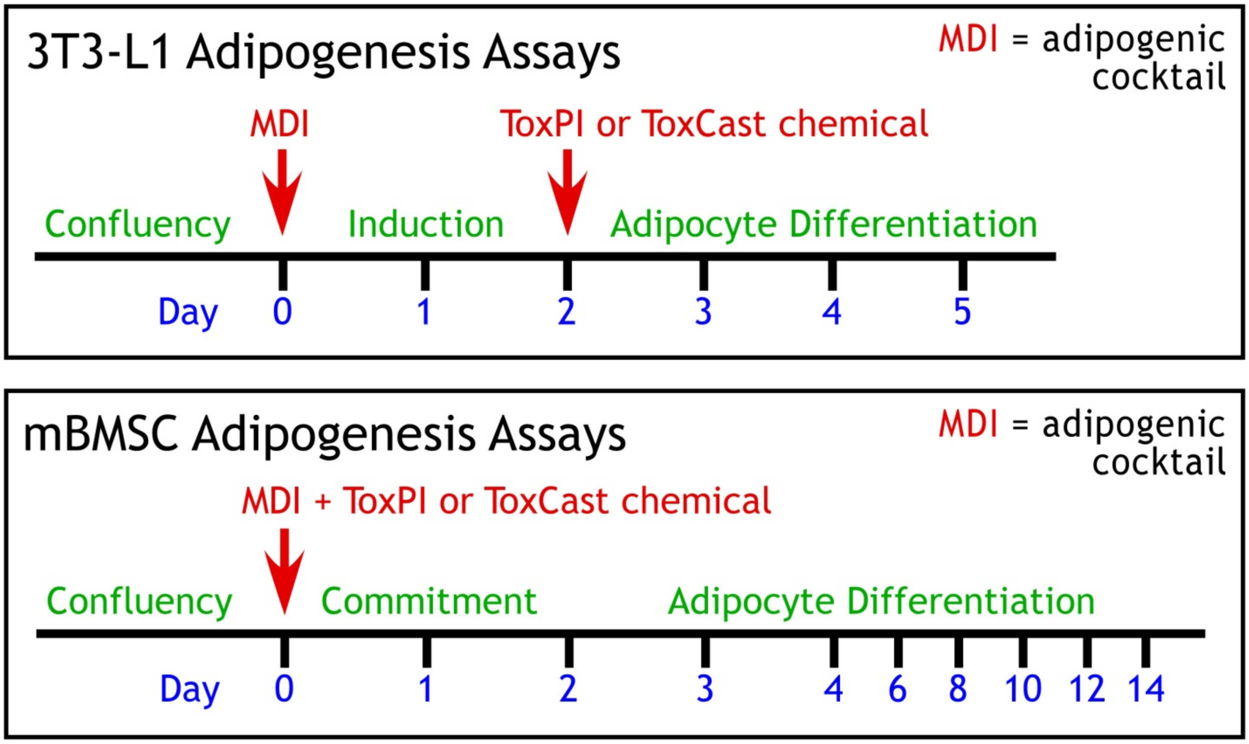

Supplemental Material, Figure S2. Schematic of adipogenesis assays. Top. 3T3-L1 cells were seeded at $2x10^4$ cells per well in 12-well plates. After 48 hours, cells were exposed to the adipogenic cocktail MDI (isobutyl-methylxanthine, dexamethasone, and insulin) for 2 days. Induction media was removed and cells were exposed to ToxCast chemicals (or controls) during 5 days replacing the media every 2 days. **Bottom.** $8x10^4$ cells/well mBMSCs were maintained as subconfluent monolayers in basic medium. Once confluent, mBMSCs were induced to differentiate with adipogenic cocktail and ToxCast chemicals (or controls) during 14 days replacing the media every 3 days.

Supplemental Material, Figure S3.

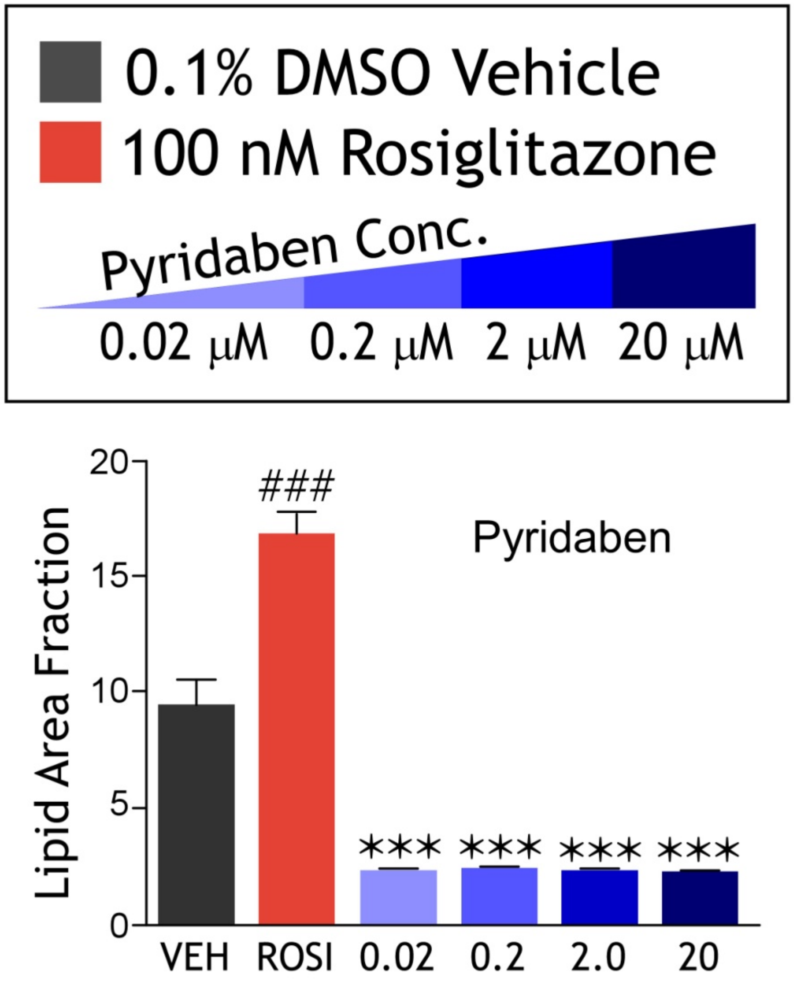

Supplemental Figure S3. ToxPi chemical pyridaben inhibits adipogenesis in 3T3-L1 preadipocytes. Adipogenesis was induced in cells according to Supplemental Material, Figure S2. Lipid accumulation was assessed by measuring the percent of surface area in each well covered by Oil Red O positive cells using Image J software. One-way ANOVA was conducted for pyridaben treatment groups and DMSO vehicle (VEH), followed by Dunnett's post-hoc test: *** $P \leqq 0.001$ compared to DMSO. Unpaired t-test was conducted for the positive control Rosiglitazone (ROSI) versus DMSO vehicle: ### $P \leqq 0.001$.

Supplemental Material, Figure S4.

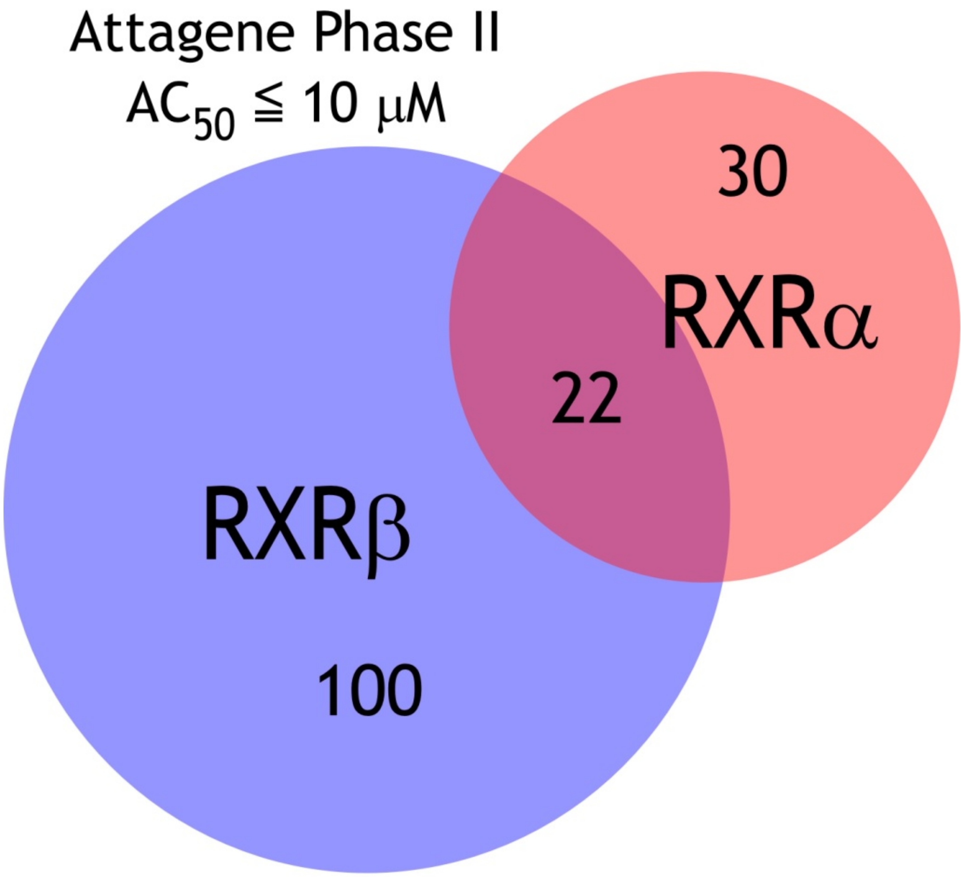

Supplemental Material, Figure S4. Attagene identifies a suspiciously large number of RXR-selective chemicals. Phase II, release 2014 (Filer et al. 2014) datasets (gain AC_{50} values) were obtained for Attagene agonist assays on RXRα and RXRβ. Chemicals scoring $AC_{50} \leqq 10$ μM for each assay were incorporated in the Venn diagrams, created by BioVenn (Hulsen et al. 2008). The Venn diagram reveals 100 RXRβ-selective and 30 RXRα-selective chemicals, with only 22 chemicals activating both receptor subtypes.

Supplemental Material, Figure S5.

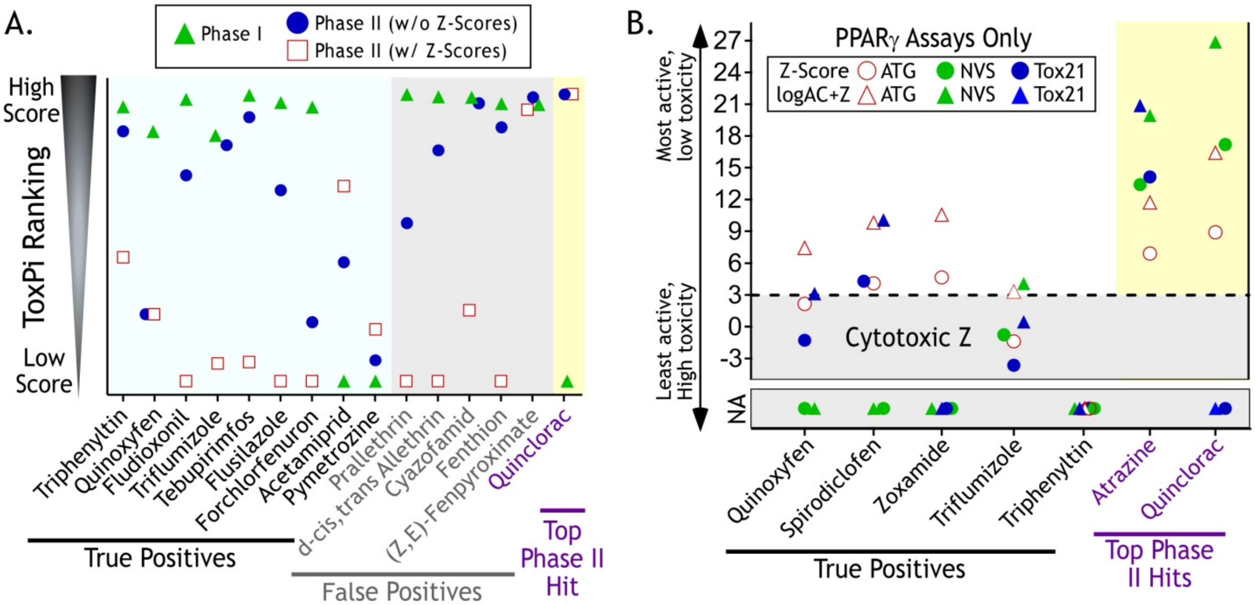

Supplemental Material, Figure S5. True positives and false positives are lost when employing ToxCast Phase II new models. (A) Green triangles: ToxPi Phase I rankings (based solely on AC_{50} values). Blue dots: ToxPi Phase II rankings using the Phase I chemical library are based solely on AC_{50} values. Squares: ToxPi Phase II rankings using Phase I chemical library were constructed by first removing chemicals with low Z-scores, and then correcting the magnitude each Pi slice by adding the Z-score to the negative log(AC_{50}). Differences between triangles and dots are due to discrepancies between Phase I and Phase II assays on the same chemical. Differences between squares and dots are attributed to the Z-score correction. **(B) Circles/dots:** Raw Z-score values derived from ToxPi Phase II data using Phase I chemical library. **Triangles:** Z-score corrected AC_{50} values derived from ToxPi Phase II data using Phase I chemical library. All true PPARγ activators rank significantly lower in both raw Z-score and Z-score corrected AC_{50} values compared to atrazine and quinclorac. However, atrazine is not a PPARγ activator in Cos7 transient transfection assays (Supplemental Material, Figure S7).

Supplemental Material, Figure S6.

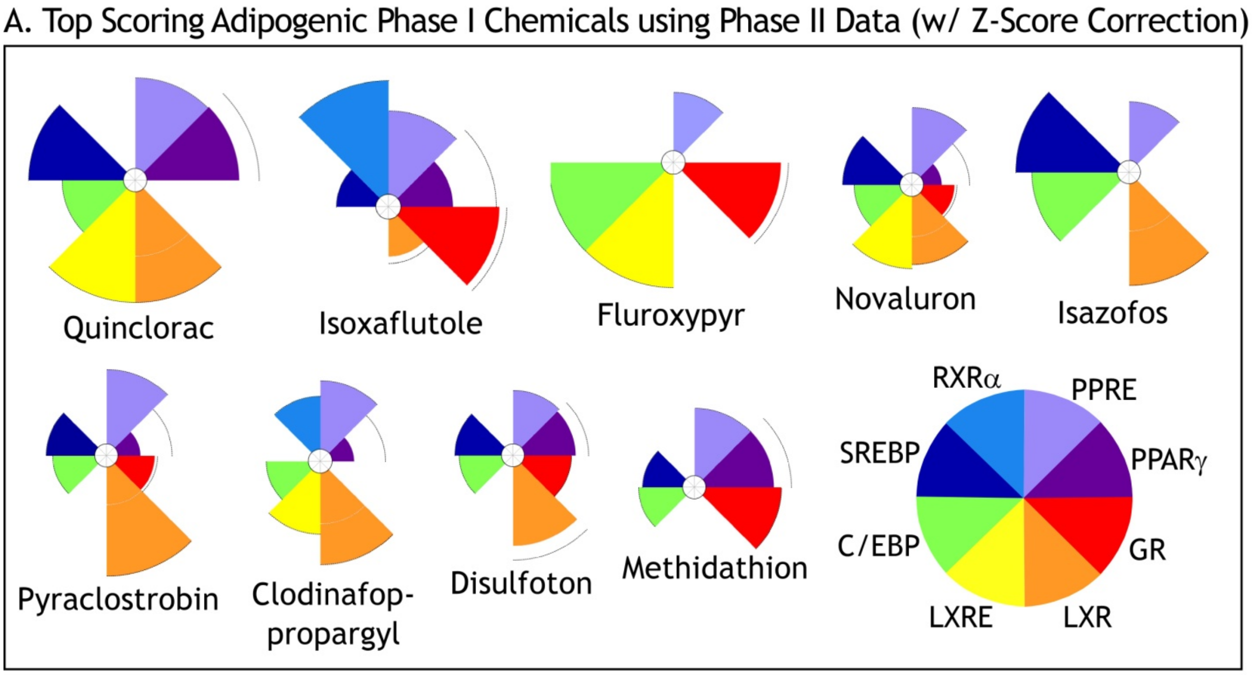

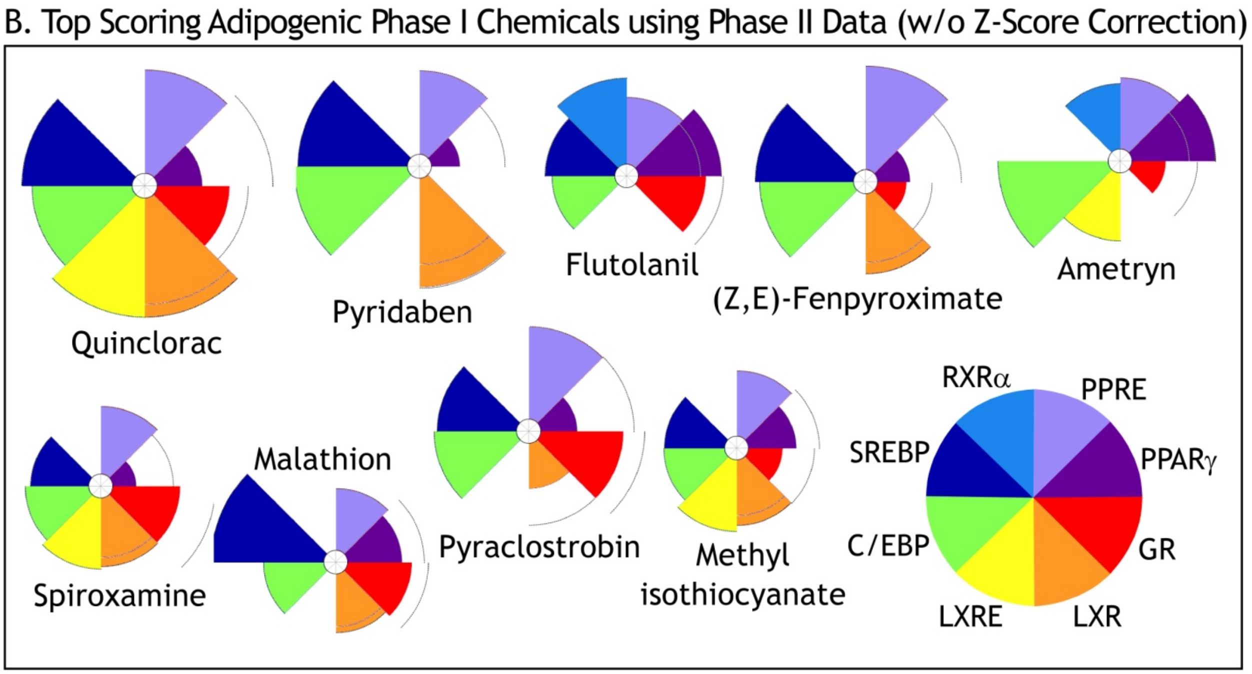

Supplemental Material, Figure S6. ToxPi regeneration using Phase II, 2014 dataset and Phase I chemical library. Adipogenesis ToxPi diagrams where slice size (magnitude) represents the activity of a ToxCast chemical in a particular assay or collection of assays (Supplemental Material, Table S1). Highest scoring ToxPi chemicals are predicted to be obesogenic. **(A)** Top scoring Phase II ToxPi chemicals with Z-score correction. The magnitude of the Pi slice is determined by adding the Z-score to the negative log(AC_{50}). **(B)** Top scoring Phase II ToxPi chemicals without Z-score correction, based solely on AC_{50} values.

Supplemental Material, Figure S7

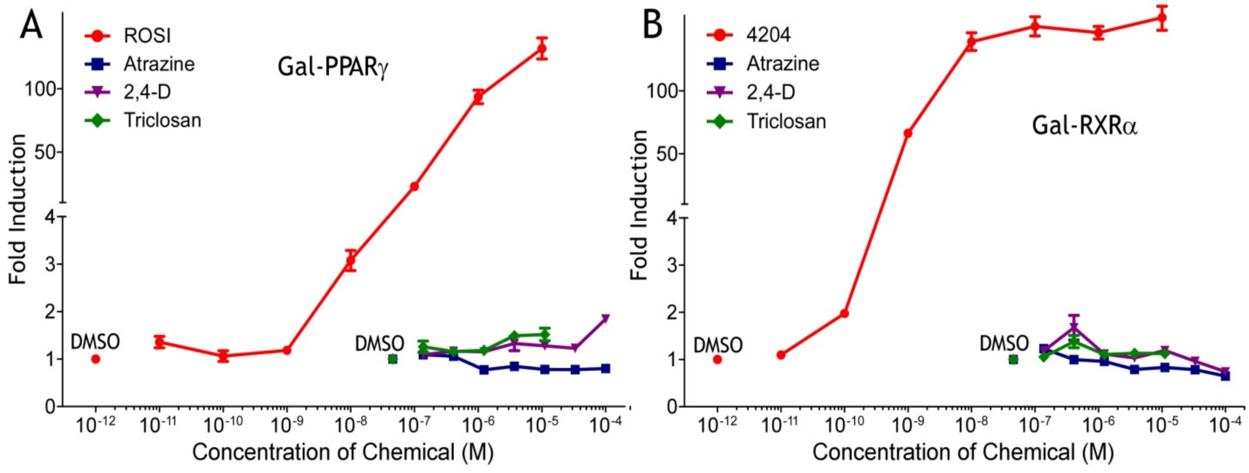

Supplemental Material, Figure S7. Phase II ToxCast Chemical Activity on PPARγ and RXRα. The ability of a graded dose series of ToxCast chemicals to activate (A) GAL4-mPPARγ or (B) GAL4-hRXRα was tested in transiently transfected Cos7 cells. (A, B) Data points are averages of triplicate transfections (3 biological replicates). Cytotoxicity, as measured by decreased ß-galactosidase activity was observed at 33 μM and 100 μM for triclosan. Data are depicted as fold induction over vehicle (0.05% DMSO) controls ± S.E.M. (A) ToxCast chemicals were tested in 3-fold serial dilutions from 100 μM through 0.137 μM, with the final data point being 0.05% DMSO. Rosiglitazone (A) and AGN1934204 (B) serve as a positive control activators and were tested in 10-fold serial dilutions.

Supplemental Material, Figure S8.

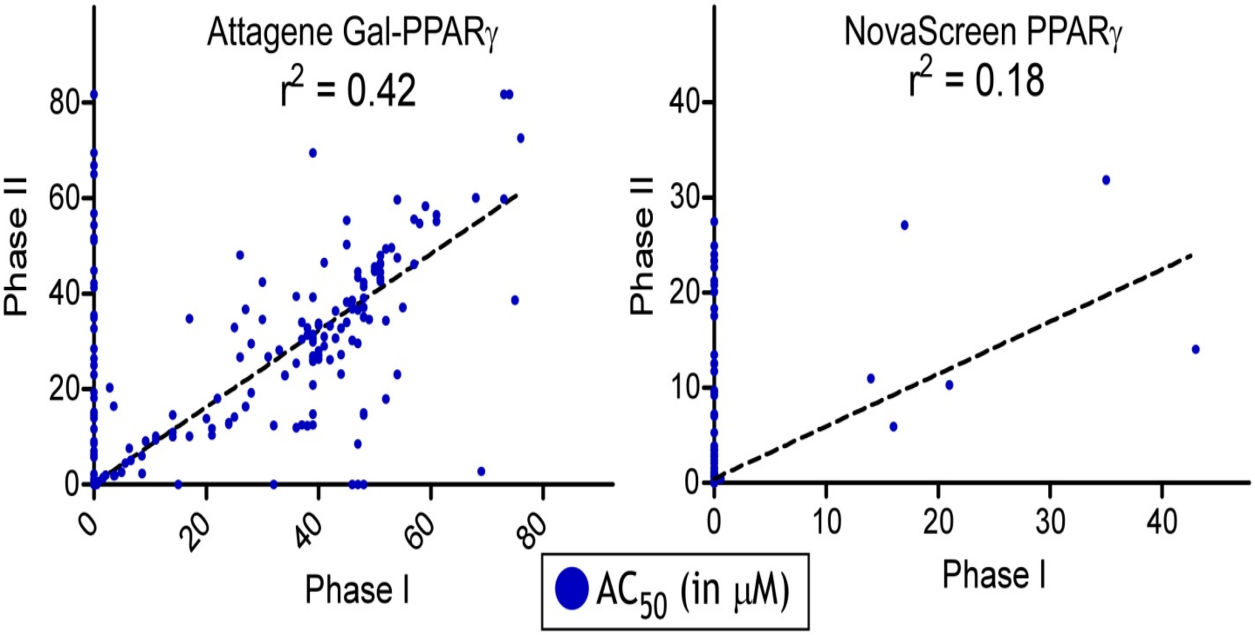

Supplemental Material, Figure S8. Linear regression analysis of ToxCast Phase I and Phase II assays on the same endpoint (same chemical/assay pair)

Table S1.

SLICE	Assay Name	Source	Assay Description	Assay Technology	Reference Compound	Cell Line	Catalog Number
PPRE	ATG_PPRE_CIS	Attagene	Factorial cis PPRE Reporter Gene Assay	In vitro (Cellular) Reporter gene assay	Rosiglitazone	HepG2	Attagene PPRE
PPARγ	ATG_PPARγ_TRANS	Attagene	Factorial trans PPARγ Reporter Gene Assay	In vitro (Cellular) Reporter gene assay	Rosiglitazone	HepG2	Attagene PPARγ
PPARγ	NCGC_PPARγ_Agonist	NCGC	GAL4 BLAM Reporter Gene Assay PPARγ Agonist	In vitro (Cellular) Reporter gene assay	Rosiglitazone	HEK293	Invitrogen SKU# K1419
PPARγ	NVS_NR_hPPARγ	Novascreen	Human PPARγ Fluorescent Ligand	Competitive Binding	Ciglitazone	NA	Caliper #100-0914
GR	NCGC_GR_Agonist	NCGC	GAL4 BLAM Reporter Gene Assay GR Agonist	In vitro (Cellular) Reporter gene assay	Dexamethasone	Hela	Invitrogen SKU# K1391
GR	NVS_NR_hGR	Novascreen	Human GR ^{3}H-Dexamethasone	Receptor Activation, Radioactivity	Triamcinolone acetonide	NA	Caliper #100-0448
GR	ATG_GR_TRANS	Attagene	Factorial trans GR Reporter Gene Assay	In vitro (Cellular) Reporter gene assay	Dexamethazone	HepG2	Attagene GR
GR	ATG_GRE_CIS	Attagene	Factorial cis GRE Reporter Gene Assay	In vitro (Cellular) Reporter gene assay	Dexamethazone	HepG2	Attagene GRE
LXR	ATG_LXRα_TRANS	Attagene	Factorial trans LXRα Reporter Gene Assay	In vitro (Cellular) Reporter gene assay	T0901317	HepG2	Attagene LXRα
LXR	ATG_LXRβ_TRANS	Attagene	Factorial trans LXRβ Reporter Gene Assay	In vitro (Cellular) Reporter gene assay	T0901317	HepG2	Attagene LXRβ
LXR	NCGC_LXR_Agonist	NCGC	GAL4 BLAM Reporter Gene Assay LXRβ Agonist	In vitro (Cellular) Reporter gene assay	T0901317	HEK293T	Invitrogen SKU# K1415
LXRE	ATG_DR4_LXR_CIS	Attagene	Factorial cis DR4/LXRE Reporter Gene Assay	In vitro (Cellular) Reporter gene assay	LXRα,β	HepG2	Attagene LXRE
CEBP	ATG_C_EBP_CIS	Attagene	Factorial cis C/EBP Reporter Gene Assay	In vitro (Cellular) Reporter gene assay	C/EBP	HepG2	Attagene C/EBP
SREBP	ATG_SREBP_CIS	Attagene	Factorial cis SREBP Reporter Gene Assay	In vitro (Cellular) Reporter gene assay	SREBP Family	HepG2	Attagene SREBP
RXRα	ATG_RXRα_TRANS	Attagene	Factorial trans RXRα Reporter Gene Assay	In vitro (Cellular) Reporter gene assay	9-cis-Retinoic acid	HepG2	Attagene RXRα
RXRα	NCGC_RXRα_Agonist	NCGC	GAL4 BLAM Reporter Gene Assay RXRα Agonist	In vitro (Cellular) Reporter gene assay	9-cis-Retinoic acid	HEK293T	Invitrogen SKU# K1411

Table S1. 16 assays were used to construct the adipogenic ToxPi models shown in Figure 3. 16 assays from Attagene, NovaScreen, and NCGC were incorporated into ToxPi models (Figure 3, Supplemental Material, Table S3). These 16 assays were chosen because they were relevant to the biological process of adipogenesis. All information provided in this table is derived from ToxCast_Phase_1_Assays_20110110.txt at http://epa.gov/ncct/toxcast/data_archive.html (2011 release; Knudsen et al. 2011). ATG_RXRα_TRANS, ATG_GR_TRANS, ATG_GRE_CIS, and NCGC_GR_Agonist assays showed no activation by any of the 320 ToxCast chemicals. The leftmost column indicates the assay or collection of assays contributing to a particular slice. For example, the PPARγ slice in Figure 3A is comprised of three assays: ATG_PPARγ_TRANS, NCGC_PPARγ_Agonist, and NVS_NR_hPPARγ.

Table S2.

			2011 Release			2014 Release			
GCID	CASRN	NAME	ATG Agonist	NVS Agonist	NCGC Agonist	ATG Agonist	NVS Agonist	NCGC Agonist	NCGC Antag.
16201	76-87-9	Triphenyltin	0.022	0.54	NA	0.02	0.28	21.6	0.02
16205	79622-59-6	Fluazinam	0.51	0.28	NA	1.2	0.26	0.07	1.3
16279	50-65-7	Niclosamide	0.56	NA	NA	0.56	NA	29.9	35.9
16322	175013-18-0	Pyraclostrobin	0.76	NA	NA	0.61	NA	NA	0.7
16377	156052-68-5	Zoxamide	1.5	NA	NA	1.3	NA	NA	0.4
16079	34256-82-1	Acetochlor	1.7	NA	NA	1.5	NA	NA	4.5
16106	23184-66-9	Butachlor	2.1	NA	NA	2.0	NA	NA	26.4
16372	68694-11-1	Triflumizole	2.8	35	NA	2.2	20.8	67.5	0.3
16305	67747-09-5	Prochloraz	3.5	NA	NA	NA	27.5	NA	81.5
16337	148477-71-8	Spirodiclofen	3.6	NA	2.8	1.9	NA	1.6	NA
16082	15972-60-8	Alachlor	3.7	43	NA	2.0	24.9	NA	3.2
16343	119168-77-3	Tebufenpyrad	4.9	NA	NA	2.6	NA	NA	23.1
16166	87674-68-8	Dimethenamid	5.6	NA	NA	4.6	NA	NA	21.4
16342	112410-23-8	Tebufenozide	6.3	NA	NA	7.6	NA	NA	50.8
16329	124495-18-7	Quinoxyfen	6.6	NA	6.7	5.1	NA	40.6	NA
16237	173584-44-6	Indoxacarb	8.5	NA	NA	6.1	NA	44.1	10.7
16199	111812-58-9	(Z,E)-Fenpyroximate	8.6	NA	NA	2.4	NA	NA	35.9
16176	28434-00-6	S-Bioallethrin	9.2	NA	NA	9.2	NA	NA	NA
16136	120116-88-3	Cyazofamid	11	NA	NA	10.1	9.6	NA	32.3
16168	110488-70-5	Dimethomorph	11	NA	NA	9.4	NA	NA	NA
16118	1897-45-6	Chlorothalonil	NA	0.6	NA	NA	0.46	82.8	0.9

Table S2. List of top scoring chemicals in ToxCast PPARγ assays. AC_{50} values (μM) from 2 PPARγ agonist assays (Attagene-ATG, NCGC), 1 PPARγ direct binding assay (Novascreen-NVS), and 1 PPARγ antagonist assay (NCGC/Tox21) are shown here, ranked on the Attagene assay -- 2011 release (Knudsen et al. 2011). GSID = DSSTox chemical identifier; CASRN = CAS Registry Number; NA = not active.

Table S3.

		Assay →	ATGC	ATGC	ATGC	ATGT	ATGT	ATGT	ATGT	ATGC	ATGT	ATGC	NCGC	NCGC	NCGC	NCGC	NVS	NVS
	CASRN	Chemical Name	CEBP	LXRE	GRE	GR	LXRα	LXRβ	PPARγ	PPRE	RXRα	SREBP	GR	LXR	PPARγ	RXRα	hGR	PPARγ
High Scoring	96182-53-5	Tebupirimfos	NA	9.4	NA	NA	30	22	43	NA	NA	31	NA	7.1	NA	NA	NA	NA
	23031-36-9	Prallethrin	29	NA	NA	NA	NA	NA	44	8.4	NA	33	NA	16.7	NA	NA	NA	NA
	584-79-2	d-cis,trans-Allethrin	25	NA	NA	NA	NA	NA	36	36	NA	48	NA	11.9	NA	NA	NA	NA
	131341-86-1	Fludioxonil	21	NA	NA	NA	NA	NA	28	24	2.72	NA	NA	NA	NA	2.7	NA	NA
	120116-88-3	Cyazofamid	8.7	NA	NA	NA	11	13	11	12	NA	NA	NA	NA	NA	NA	NA	NA
	85509-19-9	Flusilazole	NA	NA	NA	NA	NA	NA	51	46	NA	20	NA	NA	NA	NA	14	NA
	55-38-9	Fenthion	NA	34	NA	NA	33	37	36	18	NA	NA	NA	NA	NA	NA	NA	NA
	111812-58-9	Fenpyroximate (Z,E)	NA	NA	NA	NA	4.3	8.8	8.6	0.065	NA	NA	NA	6.9	NA	NA	NA	NA
	68157-60-8	Forchlorfenuron	40	NA	NA	NA	NA	37	46	26	NA	NA	NA	NA	NA	NA	NA	NA
	76-87-9	Triphenyltin	NA	NA	NA	NA	NA	NA	0.022	0.009	NA	NA	NA	NA	NA	NA	NA	NA
	119168-77-3	Tebufenpyrad	NA	NA	NA	NA	0.36	0.46	4.9	0.83	NA	NA	NA	NA	NA	NA	NA	NA
Medium Scoring	80-05-7	Bisphenol A	NA	NA	NA	NA	NA	NA	27	37	NA	NA	NA	NA	NA	NA	11	NA
	1763-23-1	PFOS	NA	NA	NA	NA	NA	NA	26	NA	NA	NA	NA	NA	NA	NA	3.5	16
	124495-18-7	Quinoxyfen	NA	NA	NA	NA	NA	NA	6.6	2.8	NA	NA	NA	NA	6.7	NA	NA	NA
	35554-44-0	Imazalil	NA	NA	NA	NA	NA	NA	73	NA	3.18	NA	NA	NA	NA	3.2	NA	NA
	96489-71-3	Pyridaben	NA	NA	NA	NA	NA	NA	14	1.6	NA	NA	NA	NA	NA	NA	NA	NA
	79622-59-6	Fluazinam	NA	NA	NA	NA	NA	NA	0.51	NA	NA	NA	NA	NA	NA	NA	NA	0.28
Inactive	135410-20-7	Acetamiprid	NA	NA	NA	NA	NA	NA	NA	NA	NA	NA	NA	NA	NA	NA	NA	NA
	3337-71-1	Asulam	NA	NA	NA	NA	NA	NA	NA	NA	NA	NA	NA	NA	NA	NA	NA	NA
	98967-40-9	Flumetsulam	NA	NA	NA	NA	NA	NA	NA	NA	NA	NA	NA	NA	NA	NA	NA	NA
	123-33-1	Maleic hydrazide	NA	NA	NA	NA	NA	NA	NA	NA	NA	NA	NA	NA	NA	NA	NA	NA
	6317-18-6	Methylene dithiocyanate	NA	NA	NA	NA	NA	NA	NA	NA	NA	NA	NA	NA	NA	NA	NA	NA
	6923-22-4	Monocrotophos	NA	NA	NA	NA	NA	NA	NA	NA	NA	NA	NA	NA	NA	NA	NA	NA
	123312-89-0	Pymetrozine	NA	NA	NA	NA	NA	NA	NA	NA	NA	NA	NA	NA	NA	NA	NA	NA

Table S3. 24 top, medium, low (zero/negative) scoring chemicals obtained by ToxPi analysis. Data directly correspond to ToxPi diagrams shown in Figure 3. Assay descriptions are available in Supplemental Material, Table S1. AC_{50} values are expressed in µM. Inactive chemicals were not active in any of the ToxCast assays for adipogenesis, feeding behavior, islet cell function, or insulin sensitivity. ATGC = Attagene cis-Factorial^{TM} assay; ATGT = Attagene trans-Factorial^{TM} assay; NCGC = GeneBLAzer agonist assay; NVS = Novascreen direct binding assay; NA = not active.

Table S4.

Gene	Primer	Sequence
36B4	Forward	AAGCGCGTCCTGGCATTGTCT
36B4	Reverse	CCGCAGGGGCAGCAGTGGT
FABP4	Forward	GTCACCATCCGGTCAGAGAG
FABP4	Reverse	TCGACTTTCCATCCCACTTC
LPL	Forward	ACAACCAGGCCTTCGAGATT
LPL	Reverse	TCAGGCCAGCTGAAGTAGGA
Fsp27	Forward	CTGGAGGAAGATGGCACAAT
Fsp27	Reverse	GGGCCACATCGATCTTCTTA

Table S4. Primer sequences.

Table S5.

A. Top Scoring Phase I PPARγ Activators (w/o Z-Score Correction)							B. Top Scoring Phase I PPARγ Activators (with Z-Score Correction)						
**PPARγ Assay**	**Attagene**		**NovaScreen**		**Tox21/NCGC**		**PPARγ Assay**	**Attagene**		**NovaScreen**		**Tox21/NCGC**	
**Chemical**	Z-Score	AC50	Z-Score	AC50	Z-Score	AC50	**Chemical**	Z-Score	AC50	Z-Score	AC50	Z-Score	AC50
Tetramethrin	5.7	0.02	1.4	0.3	-5.8	21.6	Quinclorac	8.9	0.03	17.2	0.0002	NA	NA
Hexaconazole	2.4	1.2	5.0	0.3	7.2	0.1	Atrazine	6.9	15.2	13.4	0.3	14.1	0.2
Atrazine	6.9	15.2	13.4	0.3	14.1	0.2	2-Phenylphenol	14.9	0.1	11.2	1.1	NA	NA
Flutolanil	-0.8	8.5	-1.1	10.3	-3.1	34.1	Iprodione	6.4	20.9	10.0	2.4	5.1	45.5
Ametryn	0.4	15.1	-0.8	31.8	1.1	10.2	Methidathion	5.8	30.6	15.2	0.1	NA	NA
Monocrotophos	4.0	2.2	0.3	20.8	-1.7	67.5	Triclopyr	4.8	54.7	10.2	2.1	5.3	41.5
Triclosan	1.8	8.6	2.1	7.1	0.0	25.8	Fenamiphos	5.5	35.1	6.4	21.2	7.0	14.7
Iprodione	6.4	20.9	10.0	2.4	5.1	45.5	MBP	6.6	18.2	15.2	0.1	NA	NA
MCPA	5.6	33.8	14.7	0.1	2.3	253.0	2,4-D	5.1	46.2	9.3	3.5	5.4	38.8
MEHP	-2.1	37.1	-1.1	20.1	2.2	2.8	Molinate	5.3	41.3	16.0	0.1	NA	NA
Fenamiphos	5.5	35.1	6.4	21.2	7.0	14.7	MCPA	5.6	33.8	14.7	0.1	2.3	253.0
Triclopyr	4.8	54.7	10.2	2.1	5.3	41.5	Diclosulam	6.0	26.3	NA	NA	9.6	2.9
Triflumizole	-1.4	20.4	-0.8	14.1	-3.6	79.9	Simazine	3.6	116.3	10.7	1.5	NA	NA
2,4-D	5.1	46.2	9.3	3.5	5.4	38.8	Isoxaflutole	7.5	10.4	NA	NA	6.0	26.4
2-Phenylphenol	14.9	0.1	11.2	1.1	NA	NA	Disulfoton	4.1	81.8	10.6	1.6	NA	NA
Tri-allate	0.0	29.5	10.9	0.04	NA	NA	Fluazifop-butyl	9.7	2.8	11.9	0.7	NA	NA
Trichlorfon	-0.7	58.4	NA	NA	11.0	0.05	Spirodiclofen	4.1	1.9	NA	NA	4.3	1.6
Fipronil	-0.8	39.3	10.8	0.0	NA	NA	2,4-DB	5.6	33.4	NA	NA	6.7	16.7
Methidathion	5.8	30.6	15.2	0.1	NA	NA	Acifluorfen	5.4	37.1	7.1	13.5	NA	NA
Niclosamide	-0.8	0.6	NA	NA	-7.4	29.9	Ethoprop	3.6	114.1	9.2	3.9	NA	NA

Table S5. Re-evaluation of PPARγ activators using Phase II data for three ToxCast PPARγ assays. AC_{50} values (μM) and Z-scores from ToxCast 2014 release (Filer et al. 2014) is shown. (A) The Phase I chemical library was ranked based solely on AC_{50} values (μM) of the Attagene and Tox21/NCGC PPARγ assays and NovaScreen PPARγ direct binding assay. (B) Chemicals were first filtered by removing those with cytotoxicity Z-scores less than 3 (U.S. EPA 2014) and then ranking them based on their Z-score + negative log(AC_{50}). NA = not active.

Table S6.

	PPARγ Activation (AC_{50} Values) and Adipogenesis Assays, Blumberg			PPARγ Activation Assays AC_{50} Values, 2011 Release			PPARγ Activation Assays AC_{50} Values, 2014 Release			
TOXCAST Chemical Name	Adipogenesis 3T3-L1	Activation COS7		ATG Agonist	NVS Agonist	NCGC Agonist	ATG Agonist	NVS Agonist	NCGC Agonist	NCGC Antag.
Triphenyltin	Positive	PPARγ Activator	0.02	0.022	0.54	NA	0.02	0.28	21.6	0.02
Fluazinam	Negative	PPARγ Antagonist	7.2	0.51	0.28	NA	1.2	0.26	0.07	1.3
Niclosamide	Negative	Inactive	NA	0.56	NA	NA	0.56	NA	29.9	35.9
Pyraclostrobin	Negative	Inactive	NA	0.76	NA	NA	0.61	NA	NA	0.7
Zoxamide	Positive	PPARγ Activator	1.31	1.5	NA	NA	1.3	NA	NA	0.4
Acetochlor	Negative	PPARγ Antagonist	46.7	1.7	NA	NA	1.5	NA	NA	4.5
Butachlor	Not Tested	Not Tested	NA	2.1	NA	NA	2.0	NA	NA	26.4
Triflumizole	Positive	PPARγ Activator	11.5	2.8	35	NA	2.2	20.8	67.5	0.3
Prochloraz	Negative	Inactive	NA	3.5	NA	NA	NA	27.5	NA	81.5
Spirodiclofen	Positive	PPARγ Activator	12.76	3.6	NA	2.8	1.9	NA	1.6	NA
Alachlor	Negative	PPARγ Antagonist	4.9	3.7	43	NA	2.0	24.9	NA	3.2
Tebufenpyrad	Negative	Inactive	NA	4.9	NA	NA	2.6	NA	NA	23.1
Dimethenamid	Negative	Inactive	NA	5.6	NA	NA	4.6	NA	NA	21.4
Tebufenozide	Negative	Inactive	NA	6.3	NA	NA	7.6	NA	NA	50.8
Quinoxyfen	Positive	PPARγ Activator	33.4	6.6	NA	6.7	5.1	NA	40.6	NA
Indoxacarb	Negative	Inactive	NA	8.5	NA	NA	6.1	NA	44.1	10.7
Fenpyroximate (Z,E)	Negative	Inactive	NA	8.6	NA	NA	2.4	NA	NA	35.9
S-Bioallethrin	Negative	Inactive	NA	9.2	NA	NA	9.2	NA	NA	NA
Dimethomorph	Negative	Inactive	NA	11	NA	NA	10.1	9.6	NA	32.3
Cyazofamid	Negative	Inactive	NA	11	NA	NA	9.4	NA	NA	NA
Chlorothalonil	Negative	Inactive	NA	NA	0.6	NA	NA	0.46	82.8	0.9

Table S6. Continuation of Table 1: Comparison of results in Figures 1 and 2 versus ToxCast assay data. Column 1 is a list of chemicals used in our PPARγ activation/antagonism assays and adipogenesis assays. AC_{50} values (μM) from ToxCast 2011 (Knudsen et al. 2011) and 2014 (Filer et al. 2014) releases are shown. ATG = Attagene Factorial™ PPARγ agonist assay; NVS = NovaScreen PPARγ direct binding assay; NCGC = GeneBLAzer PPARγ agonist or antagonist assay; NA = not active.

Table S7.

PPARγ Activation (AC_{50} Values) and Adipogenesis Assays, Blumberg			PPARγ Activation Assays AC_{50} Values, 2011 Release			PPARγ Activation Assays AC_{50} Values, 2014 Release				RXRα, 2011	RXR Activation Assays AC_{50} Values, 2014 Release			
Adipo-genesis	Activation		ATG Agonist	NVS Agonist	NCGC Agonist	ATG Agonist	NVS Agonist	NCGC Agonist	NCGC Antag.	ATG Agonist	ATG RXRα	ATG RXRβ	NCGC Agonist	NCGC Antag.
3T3-L1	COS7													
Positive	Inactive	NA	43	NA	NA	36.4	NA	NA	NA	NA	NA	NA	NA	NA
Negative	Inactive	NA	44	NA	NA	27.3	NA	NA	NA	NA	NA	NA	NA	NA
Negative	Inactive	NA	36	NA	NA	11.9	NA	NA	61.2	NA	NA	5.34	17.78	NA
Positive	RXRα Activator	14.3	28	NA	NA	19.3	NA	NA	27.4	2.72	23.6	1.63	2.24	NA
Negative	Inactive	NA	11	NA	NA	10.1	9.6	NA	32.3	NA	NA	NA	NA	NA
Positive	Inactive	NA	51	NA	NA	46.4	NA	NA	71.0	NA	NA	NA	NA	NA
Negative	Inactive	NA	36	NA	NA	39.4	NA	NA	NA	NA	NA	NA	NA	NA
Negative	Inactive	NA	8.6	NA	NA	2.4	NA	NA	35.9	NA	NA	NA	NA	NA
Positive	Inactive	NA	46	NA	NA	48.2	NA	NA	23.2	NA	NA	NA	NA	NA
Positive	PPARγ Activator	0.02	0.02	NA	NA	0.02	0.28	21.6	0.02	NA	NA	0.013	NA	2.51
Negative	Inactive	NA	4.9	NA	NA	2.6	NA	NA	23.1	NA	NA	NA	NA	NA
Positive	Inactive	NA	27	NA	NA	NA	NA	NA	2.4	NA	NA	NA	NA	NA
Negative	Inactive	NA	26	16	NA	26.7	5.9	NA	253.0	NA	NA	NA	NA	NA
Positive	PPARγ Activator	33.4	6.6	NA	6.7	5.1	NA	40.6	NA	NA	NA	NA	NA	39.81
Negative	Inactive	NA	73	NA	NA	59.9	NA	NA	NA	3.18	NA	NA	NA	NA
Inhibitor	PPARγ Activator	3.0	14	NA	NA	10.1	NA	NA	23.4	NA	NA	NA	NA	NA
Negative	PPARγ Antagonist	7.2	0.51	0.28	NA	1.2	0.26	0.07	1.3	NA	12.71	NA	NA	NA
Negative	Inactive	NA	NA	NA	NA	NA	NA	104.1	25.7	NA	NA	NA	NA	NA
Negative	Inactive	NA	NA	NA	NA	NA	NA	NA	NA	NA	NA	NA	NA	NA
Negative	Inactive	NA	NA	NA	NA	NA	NA	NA	NA	NA	NA	NA	NA	NA
Negative	Inactive	NA	NA	NA	NA	NA	NA	NA	NA	NA	NA	NA	NA	NA
Negative	Inactive	NA	NA	NA	NA	NA	NA	NA	NA	NA	NA	NA	NA	NA
Positive	Inactive	NA	NA	NA	NA	NA	12.5	NA	NA	NA	NA	NA	NA	NA
Positive	Inactive	NA	NA	NA	NA	NA	NA	NA	NA	NA	NA	NA	NA	NA

Table S7. Continuation of Table 2: Comparison of results in Figures 3-7 versus ToxCast Phase I assay data. Column 1 is a list of the ToxPi chemicals (Figure 3) used in PPARγ or RXRα activation assays (Figure 4) and adipogenesis assays (Figures 5-7). AC_{50} values (μM) from ToxCast 2011 (Knudsen et al. 2011) and 2014 (Filer et al. 2014) releases are shown. ATG = Attagene Factorial™ agonist assay; NVS = NovaScreen direct binding assay; NCGC = GeneBLAzer agonist or antagonist assay; NA = not active.

References

Filer D, Patisaul HB, Schug T, Reif D, Thayer K. 2014. Test driving ToxCast: endocrine profiling for 1858 chemicals included in phase II. Curr Opin Pharmacol 19: 145-152.

Hulsen T, de Vlieg J, Alkema W. 2008. BioVenn - a web application for the comparison and visualization of biological lists using area-proportional Venn diagrams. BMC Genomics 9: 488.

Knudsen TB, Houck KA, Sipes NS, Singh AV, Judson RS, Martin MT, et al. 2011. Activity profiles of 309 ToxCast chemicals evaluated across 292 biochemical targets. Toxicology 282(1-2): 1-15.

U.S. EPA. 2014. Integrated Bioactivity and Exposure Ranking: A Computational Approach for Prioritization and Screening Chemicals in the Endocrine Disruptor Screening Program. Washington, D.C. Available: http://www.regulations.gov/#!documentDetail;D=EPA-HQ-OPP-2014-0614-0003.
